# Measurement of the WZ production cross section in pp collisions at $\sqrt{s} = 7$ and 8$\,\text{TeV}$ and search for anomalous triple gauge couplings at $\sqrt{s} = 8\,\text{TeV}$

**DOI:** 10.1140/epjc/s10052-017-4730-z

**Published:** 2017-04-12

**Authors:** V. Khachatryan, A. M. Sirunyan, A. Tumasyan, W. Adam, E. Asilar, T. Bergauer, J. Brandstetter, E. Brondolin, M. Dragicevic, J. Erö, M. Flechl, M. Friedl, R. Frühwirth, V. M. Ghete, C. Hartl, N. Hörmann, J. Hrubec, M. Jeitler, A. König, I. Krätschmer, D. Liko, T. Matsushita, I. Mikulec, D. Rabady, N. Rad, B. Rahbaran, H. Rohringer, J. Schieck, J. Strauss, W. Treberer-Treberspurg, W. Waltenberger, C.-E. Wulz, V. Mossolov, N. Shumeiko, J. Suarez Gonzalez, S. Alderweireldt, E. A. De Wolf, X. Janssen, J. Lauwers, M. Van De Klundert, H. Van Haevermaet, P. Van Mechelen, N. Van Remortel, A. Van Spilbeeck, S. Abu Zeid, F. Blekman, J. D’Hondt, N. Daci, I. De Bruyn, K. Deroover, N. Heracleous, S. Lowette, S. Moortgat, L. Moreels, A. Olbrechts, Q. Python, S. Tavernier, W. Van Doninck, P. Van Mulders, I. Van Parijs, H. Brun, C. Caillol, B. Clerbaux, G. De Lentdecker, H. Delannoy, G. Fasanella, L. Favart, R. Goldouzian, A. Grebenyuk, G. Karapostoli, T. Lenzi, A. Léonard, J. Luetic, T. Maerschalk, A. Marinov, A. Randle-conde, T. Seva, C. Vander Velde, P. Vanlaer, R. Yonamine, F. Zenoni, F. Zhang, A. Cimmino, T. Cornelis, D. Dobur, A. Fagot, G. Garcia, M. Gul, D. Poyraz, S. Salva, R. Schöfbeck, M. Tytgat, W. Van Driessche, E. Yazgan, N. Zaganidis, H. Bakhshiansohi, C. Beluffi, O. Bondu, S. Brochet, G. Bruno, A. Caudron, S. De Visscher, C. Delaere, M. Delcourt, L. Forthomme, B. Francois, A. Giammanco, A. Jafari, P. Jez, M. Komm, V. Lemaitre, A. Magitteri, A. Mertens, M. Musich, C. Nuttens, K. Piotrzkowski, L. Quertenmont, M. Selvaggi, M. Vidal Marono, S. Wertz, N. Beliy, W. L. Aldá Júnior, F. L. Alves, G. A. Alves, L. Brito, C. Hensel, A. Moraes, M. E. Pol, P. Rebello Teles, E. Belchior Batista Das Chagas, W. Carvalho, J. Chinellato, A. Custódio, E. M. Da Costa, G. G. Da Silveira, D. De Jesus Damiao, C. De Oliveira Martins, S. Fonseca De Souza, L. M. Huertas Guativa, H. Malbouisson, D. Matos Figueiredo, C. Mora Herrera, L. Mundim, H. Nogima, W. L. Prado Da Silva, A. Santoro, A. Sznajder, E. J. Tonelli Manganote, A. Vilela Pereira, S. Ahuja, C. A. Bernardes, S. Dogra, T. R. Fernandez Perez Tomei, E. M. Gregores, P. G. Mercadante, C. S. Moon, S. F. Novaes, Sandra S. Padula, D. Romero Abad, J. C. Ruiz Vargas, A. Aleksandrov, R. Hadjiiska, P. Iaydjiev, M. Rodozov, S. Stoykova, G. Sultanov, M. Vutova, A. Dimitrov, I. Glushkov, L. Litov, B. Pavlov, P. Petkov, W. Fang, M. Ahmad, J. G. Bian, G. M. Chen, H. S. Chen, M. Chen, Y. Chen, T. Cheng, C. H. Jiang, D. Leggat, Z. Liu, F. Romeo, S. M. Shaheen, A. Spiezia, J. Tao, C. Wang, Z. Wang, H. Zhang, J. Zhao, Y. Ban, G. Chen, Q. Li, S. Liu, Y. Mao, S. J. Qian, D. Wang, Z. Xu, C. Avila, A. Cabrera, L. F. Chaparro Sierra, C. Florez, J. P. Gomez, C. F. González Hernández, J. D. Ruiz Alvarez, J. C. Sanabria, N. Godinovic, D. Lelas, I. Puljak, P. M. Ribeiro Cipriano, Z. Antunovic, M. Kovac, V. Brigljevic, D. Ferencek, K. Kadija, S. Micanovic, L. Sudic, T. Susa, A. Attikis, G. Mavromanolakis, J. Mousa, C. Nicolaou, F. Ptochos, P. A. Razis, H. Rykaczewski, M. Finger, M. Finger, E. Carrera Jarrin, S. Elgammal, A. Mohamed, E. Salama, B. Calpas, M. Kadastik, M. Murumaa, L. Perrini, M. Raidal, A. Tiko, C. Veelken, P. Eerola, J. Pekkanen, M. Voutilainen, J. Härkönen, V. Karimäki, R. Kinnunen, T. Lampén, K. Lassila-Perini, S. Lehti, T. Lindén, P. Luukka, T. Peltola, J. Tuominiemi, E. Tuovinen, L. Wendland, J. Talvitie, T. Tuuva, M. Besancon, F. Couderc, M. Dejardin, D. Denegri, B. Fabbro, J. L. Faure, C. Favaro, F. Ferri, S. Ganjour, S. Ghosh, A. Givernaud, P. Gras, G. Hamel de Monchenault, P. Jarry, I. Kucher, E. Locci, M. Machet, J. Malcles, J. Rander, A. Rosowsky, M. Titov, A. Zghiche, A. Abdulsalam, I. Antropov, S. Baffioni, F. Beaudette, P. Busson, L. Cadamuro, E. Chapon, C. Charlot, O. Davignon, R. Granier de Cassagnac, M. Jo, S. Lisniak, P. Miné, M. Nguyen, C. Ochando, G. Ortona, P. Paganini, P. Pigard, S. Regnard, R. Salerno, Y. Sirois, T. Strebler, Y. Yilmaz, A. Zabi, J.-L. Agram, J. Andrea, A. Aubin, D. Bloch, J.-M. Brom, M. Buttignol, E. C. Chabert, N. Chanon, C. Collard, E. Conte, X. Coubez, J.-C. Fontaine, D. Gelé, U. Goerlach, A.-C. Le Bihan, J. A. Merlin, K. Skovpen, P. Van Hove, S. Gadrat, S. Beauceron, C. Bernet, G. Boudoul, E. Bouvier, C. A. Carrillo Montoya, R. Chierici, D. Contardo, B. Courbon, P. Depasse, H. El Mamouni, J. Fan, J. Fay, S. Gascon, M. Gouzevitch, G. Grenier, B. Ille, F. Lagarde, I. B. Laktineh, M. Lethuillier, L. Mirabito, A. L. Pequegnot, S. Perries, A. Popov, D. Sabes, V. Sordini, M. Vander Donckt, P. Verdier, S. Viret, T. Toriashvili, Z. Tsamalaidze, C. Autermann, S. Beranek, L. Feld, A. Heister, M. K. Kiesel, K. Klein, M. Lipinski, A. Ostapchuk, M. Preuten, F. Raupach, S. Schael, C. Schomakers, J. F. Schulte, J. Schulz, T. Verlage, H. Weber, V. Zhukov, M. Brodski, E. Dietz-Laursonn, D. Duchardt, M. Endres, M. Erdmann, S. Erdweg, T. Esch, R. Fischer, A. Güth, M. Hamer, T. Hebbeker, C. Heidemann, K. Hoepfner, S. Knutzen, M. Merschmeyer, A. Meyer, P. Millet, S. Mukherjee, M. Olschewski, K. Padeken, T. Pook, M. Radziej, H. Reithler, M. Rieger, F. Scheuch, L. Sonnenschein, D. Teyssier, S. Thüer, V. Cherepanov, G. Flügge, W. Haj Ahmad, F. Hoehle, B. Kargoll, T. Kress, A. Künsken, J. Lingemann, A. Nehrkorn, A. Nowack, I. M. Nugent, C. Pistone, O. Pooth, A. Stahl, M. Aldaya Martin, C. Asawatangtrakuldee, K. Beernaert, O. Behnke, U. Behrens, A. A. Bin Anuar, K. Borras, A. Campbell, P. Connor, C. Contreras-Campana, F. Costanza, C. Diez Pardos, G. Dolinska, G. Eckerlin, D. Eckstein, E. Eren, E. Gallo, J. Garay Garcia, A. Geiser, A. Gizhko, J. M. Grados Luyando, P. Gunnellini, A. Harb, J. Hauk, M. Hempel, H. Jung, A. Kalogeropoulos, O. Karacheban, M. Kasemann, J. Keaveney, J. Kieseler, C. Kleinwort, I. Korol, D. Krücker, W. Lange, A. Lelek, J. Leonard, K. Lipka, A. Lobanov, W. Lohmann, R. Mankel, I.-A. Melzer-Pellmann, A. B. Meyer, G. Mittag, J. Mnich, A. Mussgiller, E. Ntomari, D. Pitzl, R. Placakyte, A. Raspereza, B. Roland, M. Ö. Sahin, P. Saxena, T. Schoerner-Sadenius, C. Seitz, S. Spannagel, N. Stefaniuk, K. D. Trippkewitz, G. P. Van Onsem, R. Walsh, C. Wissing, V. Blobel, M. Centis Vignali, A. R. Draeger, T. Dreyer, E. Garutti, K. Goebel, D. Gonzalez, J. Haller, M. Hoffmann, A. Junkes, R. Klanner, R. Kogler, N. Kovalchuk, T. Lapsien, T. Lenz, I. Marchesini, D. Marconi, M. Meyer, M. Niedziela, D. Nowatschin, J. Ott, F. Pantaleo, T. Peiffer, A. Perieanu, J. Poehlsen, C. Sander, C. Scharf, P. Schleper, A. Schmidt, S. Schumann, J. Schwandt, H. Stadie, G. Steinbrück, F. M. Stober, M. Stöver, H. Tholen, D. Troendle, E. Usai, L. Vanelderen, A. Vanhoefer, B. Vormwald, C. Barth, C. Baus, J. Berger, E. Butz, T. Chwalek, F. Colombo, W. De Boer, A. Dierlamm, S. Fink, R. Friese, M. Giffels, A. Gilbert, P. Goldenzweig, D. Haitz, F. Hartmann, S. M. Heindl, U. Husemann, I. Katkov, P. Lobelle Pardo, B. Maier, H. Mildner, M. U. Mozer, T. Müller, Th. Müller, M. Plagge, G. Quast, K. Rabbertz, S. Röcker, F. Roscher, M. Schröder, I. Shvetsov, G. Sieber, H. J. Simonis, R. Ulrich, J. Wagner-Kuhr, S. Wayand, M. Weber, T. Weiler, S. Williamson, C. Wöhrmann, R. Wolf, G. Anagnostou, G. Daskalakis, T. Geralis, V. A. Giakoumopoulou, A. Kyriakis, D. Loukas, I. Topsis-Giotis, A. Agapitos, S. Kesisoglou, A. Panagiotou, N. Saoulidou, E. Tziaferi, I. Evangelou, G. Flouris, C. Foudas, P. Kokkas, N. Loukas, N. Manthos, I. Papadopoulos, E. Paradas, N. Filipovic, G. Bencze, C. Hajdu, P. Hidas, D. Horvath, F. Sikler, V. Veszpremi, G. Vesztergombi, A. J. Zsigmond, N. Beni, S. Czellar, J. Karancsi, A. Makovec, J. Molnar, Z. Szillasi, M. Bartók, P. Raics, Z. L. Trocsanyi, B. Ujvari, S. Bahinipati, S. Choudhury, P. Mal, K. Mandal, A. Nayak, D. K. Sahoo, N. Sahoo, S. K. Swain, S. Bansal, S. B. Beri, V. Bhatnagar, R. Chawla, U.Bhawandeep, A. K. Kalsi, A. Kaur, M. Kaur, R. Kumar, A. Mehta, M. Mittal, J. B. Singh, G. Walia, Ashok Kumar, A. Bhardwaj, B. C. Choudhary, R. B. Garg, S. Keshri, S. Malhotra, M. Naimuddin, N. Nishu, K. Ranjan, R. Sharma, V. Sharma, R. Bhattacharya, S. Bhattacharya, K. Chatterjee, S. Dey, S. Dutt, S. Dutta, S. Ghosh, N. Majumdar, A. Modak, K. Mondal, S. Mukhopadhyay, S. Nandan, A. Purohit, A. Roy, D. Roy, S. Roy Chowdhury, S. Sarkar, M. Sharan, S. Thakur, P. K. Behera, R. Chudasama, D. Dutta, V. Jha, V. Kumar, A. K. Mohanty, P. K. Netrakanti, L. M. Pant, P. Shukla, A. Topkar, T. Aziz, S. Dugad, G. Kole, B. Mahakud, S. Mitra, G. B. Mohanty, B. Parida, N. Sur, B. Sutar, S. Banerjee, S. Bhowmik, R. K. Dewanjee, S. Ganguly, M. Guchait, Sa. Jain, S. Kumar, M. Maity, G. Majumder, K. Mazumdar, T. Sarkar, N. Wickramage, S. Chauhan, S. Dube, V. Hegde, A. Kapoor, K. Kothekar, A. Rane, S. Sharma, H. Behnamian, S. Chenarani, E. Eskandari Tadavani, S. M. Etesami, A. Fahim, M. Khakzad, M. Mohammadi Najafabadi, M. Naseri, S. Paktinat Mehdiabadi, F. Rezaei Hosseinabadi, B. Safarzadeh, M. Zeinali, M. Felcini, M. Grunewald, M. Abbrescia, C. Calabria, C. Caputo, A. Colaleo, D. Creanza, L. Cristella, N. De Filippis, M. De Palma, L. Fiore, G. Iaselli, G. Maggi, M. Maggi, G. Miniello, S. My, S. Nuzzo, A. Pompili, G. Pugliese, R. Radogna, A. Ranieri, G. Selvaggi, L. Silvestris, R. Venditti, P. Verwilligen, G. Abbiendi, C. Battilana, D. Bonacorsi, S. Braibant-Giacomelli, L. Brigliadori, R. Campanini, P. Capiluppi, A. Castro, F. R. Cavallo, S. S. Chhibra, G. Codispoti, M. Cuffiani, G. M. Dallavalle, F. Fabbri, A. Fanfani, D. Fasanella, P. Giacomelli, C. Grandi, L. Guiducci, S. Marcellini, G. Masetti, A. Montanari, F. L. Navarria, A. Perrotta, A. M. Rossi, T. Rovelli, G. P. Siroli, N. Tosi, S. Albergo, M. Chiorboli, S. Costa, A. Di Mattia, F. Giordano, R. Potenza, A. Tricomi, C. Tuve, G. Barbagli, V. Ciulli, C. Civinini, R. D’Alessandro, E. Focardi, V. Gori, P. Lenzi, M. Meschini, S. Paoletti, G. Sguazzoni, L. Viliani, L. Benussi, S. Bianco, F. Fabbri, D. Piccolo, F. Primavera, V. Calvelli, F. Ferro, M. Lo Vetere, M. R. Monge, E. Robutti, S. Tosi, L. Brianza, M. E. Dinardo, S. Fiorendi, S. Gennai, A. Ghezzi, P. Govoni, S. Malvezzi, R. A. Manzoni, B. Marzocchi, D. Menasce, L. Moroni, M. Paganoni, D. Pedrini, S. Pigazzini, S. Ragazzi, T. Tabarelli de Fatis, S. Buontempo, N. Cavallo, G. De Nardo, S. Di Guida, M. Esposito, F. Fabozzi, A. O. M. Iorio, G. Lanza, L. Lista, S. Meola, P. Paolucci, C. Sciacca, F. Thyssen, P. Azzi, N. Bacchetta, L. Benato, D. Bisello, A. Boletti, R. Carlin, A. Carvalho Antunes De Oliveira, P. Checchia, M. Dall’Osso, P. De Castro Manzano, T. Dorigo, U. Dosselli, F. Gasparini, U. Gasparini, A. Gozzelino, S. Lacaprara, M. Margoni, A. T. Meneguzzo, J. Pazzini, N. Pozzobon, P. Ronchese, F. Simonetto, E. Torassa, M. Zanetti, P. Zotto, A. Zucchetta, G. Zumerle, A. Braghieri, A. Magnani, P. Montagna, S. P. Ratti, V. Re, C. Riccardi, P. Salvini, I. Vai, P. Vitulo, L. Alunni Solestizi, G. M. Bilei, D. Ciangottini, L. Fanò, P. Lariccia, R. Leonardi, G. Mantovani, M. Menichelli, A. Saha, A. Santocchia, K. Androsov, P. Azzurri, G. Bagliesi, J. Bernardini, T. Boccali, R. Castaldi, M. A. Ciocci, R. Dell’Orso, S. Donato, G. Fedi, A. Giassi, M. T. Grippo, F. Ligabue, T. Lomtadze, L. Martini, A. Messineo, F. Palla, A. Rizzi, A. Savoy-Navarro, P. Spagnolo, R. Tenchini, G. Tonelli, A. Venturi, P. G. Verdini, L. Barone, F. Cavallari, M. Cipriani, G. D’imperio, D. Del Re, M. Diemoz, S. Gelli, C. Jorda, E. Longo, F. Margaroli, P. Meridiani, G. Organtini, R. Paramatti, F. Preiato, S. Rahatlou, C. Rovelli, F. Santanastasio, N. Amapane, R. Arcidiacono, S. Argiro, M. Arneodo, N. Bartosik, R. Bellan, C. Biino, N. Cartiglia, F. Cenna, M. Costa, R. Covarelli, A. Degano, N. Demaria, L. Finco, B. Kiani, C. Mariotti, S. Maselli, E. Migliore, V. Monaco, E. Monteil, M. M. Obertino, L. Pacher, N. Pastrone, M. Pelliccioni, G. L. Pinna Angioni, F. Ravera, A. Romero, M. Ruspa, R. Sacchi, K. Shchelina, V. Sola, A. Solano, A. Staiano, P. Traczyk, S. Belforte, M. Casarsa, F. Cossutti, G. Della Ricca, C. La Licata, A. Schizzi, A. Zanetti, D. H. Kim, G. N. Kim, M. S. Kim, S. Lee, S. W. Lee, Y. D. Oh, S. Sekmen, D. C. Son, Y. C. Yang, A. Lee, J. A. Brochero Cifuentes, T. J. Kim, S. Cho, S. Choi, Y. Go, D. Gyun, S. Ha, B. Hong, Y. Jo, Y. Kim, B. Lee, K. Lee, K. S. Lee, S. Lee, J. Lim, S. K. Park, Y. Roh, J. Almond, J. Kim, S. B. Oh, S. h. Seo, U. K. Yang, H. D. Yoo, G. B. Yu, M. Choi, H. Kim, H. Kim, J. H. Kim, J. S. H. Lee, I. C. Park, G. Ryu, M. S. Ryu, Y. Choi, J. Goh, C. Hwang, J. Lee, I. Yu, V. Dudenas, A. Juodagalvis, J. Vaitkus, I. Ahmed, Z. A. Ibrahim, J. R. Komaragiri, M. A. B. Md Ali, F. Mohamad Idris, W. A. T. Wan Abdullah, M. N. Yusli, Z. Zolkapli, H. Castilla-Valdez, E. De La Cruz-Burelo, I. Heredia-De La Cruz, A. Hernandez-Almada, R. Lopez-Fernandez, R. Magaña Villalba, J. Mejia Guisao, A. Sanchez-Hernandez, S. Carrillo Moreno, C. Oropeza Barrera, F. Vazquez Valencia, S. Carpinteyro, I. Pedraza, H. A. Salazar Ibarguen, C. Uribe Estrada, A. Morelos Pineda, D. Krofcheck, P. H. Butler, A. Ahmad, M. Ahmad, Q. Hassan, H. R. Hoorani, W. A. Khan, M. A. Shah, M. Shoaib, M. Waqas, H. Bialkowska, M. Bluj, B. Boimska, T. Frueboes, M. Górski, M. Kazana, K. Nawrocki, K. Romanowska-Rybinska, M. Szleper, P. Zalewski, K. Bunkowski, A. Byszuk, K. Doroba, A. Kalinowski, M. Konecki, J. Krolikowski, M. Misiura, M. Olszewski, M. Walczak, P. Bargassa, C. Beirão Da Cruz E Silva, A. Di Francesco, P. Faccioli, P. G. Ferreira Parracho, M. Gallinaro, J. Hollar, N. Leonardo, L. Lloret Iglesias, M. V. Nemallapudi, J. Rodrigues Antunes, J. Seixas, O. Toldaiev, D. Vadruccio, J. Varela, P. Vischia, P. Bunin, A. Golunov, I. Golutvin, N. Gorbounov, V. Karjavin, V. Korenkov, A. Lanev, A. Malakhov, V. Matveev, V. V. Mitsyn, P. Moisenz, V. Palichik, V. Perelygin, S. Shmatov, S. Shulha, N. Skatchkov, V. Smirnov, E. Tikhonenko, A. Zarubin, L. Chtchipounov, V. Golovtsov, Y. Ivanov, V. Kim, E. Kuznetsova, V. Murzin, V. Oreshkin, V. Sulimov, A. Vorobyev, Yu. Andreev, A. Dermenev, S. Gninenko, N. Golubev, A. Karneyeu, M. Kirsanov, N. Krasnikov, A. Pashenkov, D. Tlisov, A. Toropin, V. Epshteyn, V. Gavrilov, N. Lychkovskaya, V. Popov, I. Pozdnyakov, G. Safronov, A. Spiridonov, M. Toms, E. Vlasov, A. Zhokin, A. Bylinkin, R. Chistov, M. Danilov, V. Rusinov, V. Andreev, M. Azarkin, I. Dremin, M. Kirakosyan, A. Leonidov, S. V. Rusakov, A. Terkulov, A. Baskakov, A. Belyaev, E. Boos, M. Dubinin, L. Dudko, A. Ershov, A. Gribushin, V. Klyukhin, O. Kodolova, I. Lokhtin, I. Miagkov, S. Obraztsov, S. Petrushanko, V. Savrin, A. Snigirev, V. Blinov, Y. Skovpen, I. Azhgirey, I. Bayshev, S. Bitioukov, D. Elumakhov, V. Kachanov, A. Kalinin, D. Konstantinov, V. Krychkine, V. Petrov, R. Ryutin, A. Sobol, S. Troshin, N. Tyurin, A. Uzunian, A. Volkov, P. Adzic, P. Cirkovic, D. Devetak, M. Dordevic, J. Milosevic, V. Rekovic, J. Alcaraz Maestre, M. Barrio Luna, E. Calvo, M. Cerrada, M. Chamizo Llatas, N. Colino, B. De La Cruz, A. Delgado Peris, A. Escalante Del Valle, C. Fernandez Bedoya, J. P. Fernández Ramos, J. Flix, M. C. Fouz, P. Garcia-Abia, O. Gonzalez Lopez, S. Goy Lopez, J. M. Hernandez, M. I. Josa, E. Navarro De Martino, A. Pérez-Calero Yzquierdo, J. Puerta Pelayo, A. Quintario Olmeda, I. Redondo, L. Romero, M. S. Soares, J. F. de Trocóniz, M. Missiroli, D. Moran, J. Cuevas, J. Fernandez Menendez, I. Gonzalez Caballero, J. R. González Fernández, E. Palencia Cortezon, S. Sanchez Cruz, I. Suárez Andrés, J. M. Vizan Garcia, I. J. Cabrillo, A. Calderon, J. R. Castiñeiras De Saa, E. Curras, M. Fernandez, J. Garcia-Ferrero, G. Gomez, A. Lopez Virto, J. Marco, C. Martinez Rivero, F. Matorras, J. Piedra Gomez, T. Rodrigo, A. Ruiz-Jimeno, L. Scodellaro, N. Trevisani, I. Vila, R. Vilar Cortabitarte, D. Abbaneo, E. Auffray, G. Auzinger, M. Bachtis, P. Baillon, A. H. Ball, D. Barney, P. Bloch, A. Bocci, A. Bonato, C. Botta, T. Camporesi, R. Castello, M. Cepeda, G. Cerminara, M. D’Alfonso, D. d’Enterria, A. Dabrowski, V. Daponte, A. David, M. De Gruttola, F. De Guio, A. De Roeck, E. Di Marco, M. Dobson, B. Dorney, T. du Pree, D. Duggan, M. Dünser, N. Dupont, A. Elliott-Peisert, S. Fartoukh, G. Franzoni, J. Fulcher, W. Funk, D. Gigi, K. Gill, M. Girone, F. Glege, D. Gulhan, S. Gundacker, M. Guthoff, J. Hammer, P. Harris, J. Hegeman, V. Innocente, P. Janot, H. Kirschenmann, V. Knünz, A. Kornmayer, M. J. Kortelainen, K. Kousouris, M. Krammer, P. Lecoq, C. Lourenço, M. T. Lucchini, L. Malgeri, M. Mannelli, A. Martelli, F. Meijers, S. Mersi, E. Meschi, F. Moortgat, S. Morovic, M. Mulders, H. Neugebauer, S. Orfanelli, L. Orsini, L. Pape, E. Perez, M. Peruzzi, A. Petrilli, G. Petrucciani, A. Pfeiffer, M. Pierini, A. Racz, T. Reis, G. Rolandi, M. Rovere, M. Ruan, H. Sakulin, J. B. Sauvan, C. Schäfer, C. Schwick, M. Seidel, A. Sharma, P. Silva, M. Simon, P. Sphicas, J. Steggemann, M. Stoye, Y. Takahashi, M. Tosi, D. Treille, A. Triossi, A. Tsirou, V. Veckalns, G. I. Veres, N. Wardle, H. K. Wöhri, A. Zagozdzinska, W. D. Zeuner, W. Bertl, K. Deiters, W. Erdmann, R. Horisberger, Q. Ingram, H. C. Kaestli, D. Kotlinski, U. Langenegger, T. Rohe, F. Bachmair, L. Bäni, L. Bianchini, B. Casal, G. Dissertori, M. Dittmar, M. Donegà, P. Eller, C. Grab, C. Heidegger, D. Hits, J. Hoss, G. Kasieczka, P. Lecomte, W. Lustermann, B. Mangano, M. Marionneau, P. Martinez Ruiz del Arbol, M. Masciovecchio, M. T. Meinhard, D. Meister, F. Micheli, P. Musella, F. Nessi-Tedaldi, F. Pandolfi, J. Pata, F. Pauss, G. Perrin, L. Perrozzi, M. Quittnat, M. Rossini, M. Schönenberger, A. Starodumov, V. R. Tavolaro, K. Theofilatos, R. Wallny, T. K. Aarrestad, C. Amsler, L. Caminada, M. F. Canelli, A. De Cosa, C. Galloni, A. Hinzmann, T. Hreus, B. Kilminster, C. Lange, J. Ngadiuba, D. Pinna, G. Rauco, P. Robmann, D. Salerno, Y. Yang, V. Candelise, T. H. Doan, Sh. Jain, R. Khurana, M. Konyushikhin, C. M. Kuo, W. Lin, Y. J. Lu, A. Pozdnyakov, S. S. Yu, Arun Kumar, P. Chang, Y. H. Chang, Y. W. Chang, Y. Chao, K. F. Chen, P. H. Chen, C. Dietz, F. Fiori, W.-S. Hou, Y. Hsiung, Y. F. Liu, R.-S. Lu, M. Miñano Moya, E. Paganis, A. Psallidas, J. F. Tsai, Y. M. Tzeng, B. Asavapibhop, G. Singh, N. Srimanobhas, N. Suwonjandee, A. Adiguzel, S. Damarseckin, Z. S. Demiroglu, C. Dozen, E. Eskut, S. Girgis, G. Gokbulut, Y. Guler, E. Gurpinar, I. Hos, E. E. Kangal, O. Kara, U. Kiminsu, M. Oglakci, G. Onengut, K. Ozdemir, S. Ozturk, A. Polatoz, D. Sunar Cerci, B. Tali, S. Turkcapar, I. S. Zorbakir, C. Zorbilmez, B. Bilin, S. Bilmis, B. Isildak, G. Karapinar, M. Yalvac, M. Zeyrek, E. Gülmez, M. Kaya, O. Kaya, E. A. Yetkin, T. Yetkin, A. Cakir, K. Cankocak, S. Sen, B. Grynyov, L. Levchuk, P. Sorokin, R. Aggleton, F. Ball, L. Beck, J. J. Brooke, D. Burns, E. Clement, D. Cussans, H. Flacher, J. Goldstein, M. Grimes, G. P. Heath, H. F. Heath, J. Jacob, L. Kreczko, C. Lucas, D. M. Newbold, S. Paramesvaran, A. Poll, T. Sakuma, S. Seif El Nasr-storey, D. Smith, V. J. Smith, K. W. Bell, A. Belyaev, C. Brew, R. M. Brown, L. Calligaris, D. Cieri, D. J. A. Cockerill, J. A. Coughlan, K. Harder, S. Harper, E. Olaiya, D. Petyt, C. H. Shepherd-Themistocleous, A. Thea, I. R. Tomalin, T. Williams, M. Baber, R. Bainbridge, O. Buchmuller, A. Bundock, D. Burton, S. Casasso, M. Citron, D. Colling, L. Corpe, P. Dauncey, G. Davies, A. De Wit, M. Della Negra, R. Di Maria, P. Dunne, A. Elwood, D. Futyan, Y. Haddad, G. Hall, G. Iles, T. James, R. Lane, C. Laner, R. Lucas, L. Lyons, A.-M. Magnan, S. Malik, L. Mastrolorenzo, J. Nash, A. Nikitenko, J. Pela, B. Penning, M. Pesaresi, D. M. Raymond, A. Richards, A. Rose, C. Seez, S. Summers, A. Tapper, K. Uchida, M. Vazquez Acosta, T. Virdee, J. Wright, S. C. Zenz, J. E. Cole, P. R. Hobson, A. Khan, P. Kyberd, D. Leslie, I. D. Reid, P. Symonds, L. Teodorescu, M. Turner, A. Borzou, K. Call, J. Dittmann, K. Hatakeyama, H. Liu, N. Pastika, O. Charaf, S. I. Cooper, C. Henderson, P. Rumerio, D. Arcaro, A. Avetisyan, T. Bose, D. Gastler, D. Rankin, C. Richardson, J. Rohlf, L. Sulak, D. Zou, G. Benelli, E. Berry, D. Cutts, A. Garabedian, J. Hakala, U. Heintz, J. M. Hogan, O. Jesus, E. Laird, G. Landsberg, Z. Mao, M. Narain, S. Piperov, S. Sagir, E. Spencer, R. Syarif, R. Breedon, G. Breto, D. Burns, M. Calderon De La Barca Sanchez, S. Chauhan, M. Chertok, J. Conway, R. Conway, P. T. Cox, R. Erbacher, C. Flores, G. Funk, M. Gardner, W. Ko, R. Lander, C. Mclean, M. Mulhearn, D. Pellett, J. Pilot, F. Ricci-Tam, S. Shalhout, J. Smith, M. Squires, D. Stolp, M. Tripathi, S. Wilbur, R. Yohay, R. Cousins, P. Everaerts, A. Florent, J. Hauser, M. Ignatenko, D. Saltzberg, E. Takasugi, V. Valuev, M. Weber, K. Burt, R. Clare, J. Ellison, J. W. Gary, G. Hanson, J. Heilman, P. Jandir, E. Kennedy, F. Lacroix, O. R. Long, M. Malberti, M. Olmedo Negrete, M. I. Paneva, A. Shrinivas, H. Wei, S. Wimpenny, B. R. Yates, J. G. Branson, G. B. Cerati, S. Cittolin, M. Derdzinski, R. Gerosa, A. Holzner, D. Klein, V. Krutelyov, J. Letts, I. Macneill, D. Olivito, S. Padhi, M. Pieri, M. Sani, V. Sharma, S. Simon, M. Tadel, A. Vartak, S. Wasserbaech, C. Welke, J. Wood, F. Würthwein, A. Yagil, G. Zevi Della Porta, R. Bhandari, J. Bradmiller-Feld, C. Campagnari, A. Dishaw, V. Dutta, K. Flowers, M. Franco Sevilla, P. Geffert, C. George, F. Golf, L. Gouskos, J. Gran, R. Heller, J. Incandela, N. Mccoll, S. D. Mullin, A. Ovcharova, J. Richman, D. Stuart, I. Suarez, C. West, J. Yoo, D. Anderson, A. Apresyan, J. Bendavid, A. Bornheim, J. Bunn, Y. Chen, J. Duarte, J. M. Lawhorn, A. Mott, H. B. Newman, C. Pena, M. Spiropulu, J. R. Vlimant, S. Xie, R. Y. Zhu, M. B. Andrews, V. Azzolini, B. Carlson, T. Ferguson, M. Paulini, J. Russ, M. Sun, H. Vogel, I. Vorobiev, J. P. Cumalat, W. T. Ford, F. Jensen, A. Johnson, M. Krohn, T. Mulholland, K. Stenson, S. R. Wagner, J. Alexander, J. Chaves, J. Chu, S. Dittmer, K. Mcdermott, N. Mirman, G. Nicolas Kaufman, J. R. Patterson, A. Rinkevicius, A. Ryd, L. Skinnari, L. Soffi, S. M. Tan, Z. Tao, J. Thom, J. Tucker, P. Wittich, M. Zientek, D. Winn, S. Abdullin, M. Albrow, G. Apollinari, S. Banerjee, L. A. T. Bauerdick, A. Beretvas, J. Berryhill, P. C. Bhat, G. Bolla, K. Burkett, J. N. Butler, H. W. K. Cheung, F. Chlebana, S. Cihangir, M. Cremonesi, V. D. Elvira, I. Fisk, J. Freeman, E. Gottschalk, L. Gray, D. Green, S. Grünendahl, O. Gutsche, D. Hare, R. M. Harris, S. Hasegawa, J. Hirschauer, Z. Hu, B. Jayatilaka, S. Jindariani, M. Johnson, U. Joshi, B. Klima, B. Kreis, S. Lammel, J. Linacre, D. Lincoln, R. Lipton, T. Liu, R. Lopes De Sá, J. Lykken, K. Maeshima, N. Magini, J. M. Marraffino, S. Maruyama, D. Mason, P. McBride, P. Merkel, S. Mrenna, S. Nahn, C. Newman-Holmes, V. O’Dell, K. Pedro, O. Prokofyev, G. Rakness, L. Ristori, E. Sexton-Kennedy, A. Soha, W. J. Spalding, L. Spiegel, S. Stoynev, N. Strobbe, L. Taylor, S. Tkaczyk, N. V. Tran, L. Uplegger, E. W. Vaandering, C. Vernieri, M. Verzocchi, R. Vidal, M. Wang, H. A. Weber, A. Whitbeck, D. Acosta, P. Avery, P. Bortignon, D. Bourilkov, A. Brinkerhoff, A. Carnes, M. Carver, D. Curry, S. Das, R. D. Field, I. K. Furic, J. Konigsberg, A. Korytov, P. Ma, K. Matchev, H. Mei, P. Milenovic, G. Mitselmakher, D. Rank, L. Shchutska, D. Sperka, L. Thomas, J. Wang, S. Wang, J. Yelton, S. Linn, P. Markowitz, G. Martinez, J. L. Rodriguez, A. Ackert, J. R. Adams, T. Adams, A. Askew, S. Bein, B. Diamond, S. Hagopian, V. Hagopian, K. F. Johnson, A. Khatiwada, H. Prosper, A. Santra, M. Weinberg, M. M. Baarmand, V. Bhopatkar, S. Colafranceschi, M. Hohlmann, D. Noonan, T. Roy, F. Yumiceva, M. R. Adams, L. Apanasevich, D. Berry, R. R. Betts, I. Bucinskaite, R. Cavanaugh, O. Evdokimov, L. Gauthier, C. E. Gerber, D. J. Hofman, P. Kurt, C. O’Brien, I. D. Sandoval Gonzalez, P. Turner, N. Varelas, H. Wang, Z. Wu, M. Zakaria, J. Zhang, B. Bilki, W. Clarida, K. Dilsiz, S. Durgut, R. P. Gandrajula, M. Haytmyradov, V. Khristenko, J.-P. Merlo, H. Mermerkaya, A. Mestvirishvili, A. Moeller, J. Nachtman, H. Ogul, Y. Onel, F. Ozok, A. Penzo, C. Snyder, E. Tiras, J. Wetzel, K. Yi, I. Anderson, B. Blumenfeld, A. Cocoros, N. Eminizer, D. Fehling, L. Feng, A. V. Gritsan, P. Maksimovic, M. Osherson, J. Roskes, U. Sarica, M. Swartz, M. Xiao, Y. Xin, C. You, A. Al-bataineh, P. Baringer, A. Bean, J. Bowen, C. Bruner, J. Castle, R. P. Kenny, A. Kropivnitskaya, D. Majumder, W. Mcbrayer, M. Murray, S. Sanders, R. Stringer, J. D. Tapia Takaki, Q. Wang, A. Ivanov, K. Kaadze, S. Khalil, M. Makouski, Y. Maravin, A. Mohammadi, L. K. Saini, N. Skhirtladze, S. Toda, D. Lange, F. Rebassoo, D. Wright, C. Anelli, A. Baden, O. Baron, A. Belloni, B. Calvert, S. C. Eno, C. Ferraioli, J. A. Gomez, N. J. Hadley, S. Jabeen, R. G. Kellogg, T. Kolberg, J. Kunkle, Y. Lu, A. C. Mignerey, Y. H. Shin, A. Skuja, M. B. Tonjes, S. C. Tonwar, D. Abercrombie, B. Allen, A. Apyan, R. Barbieri, A. Baty, R. Bi, K. Bierwagen, S. Brandt, W. Busza, I. A. Cali, Z. Demiragli, L. Di Matteo, G. Gomez Ceballos, M. Goncharov, D. Hsu, Y. Iiyama, G. M. Innocenti, M. Klute, D. Kovalskyi, K. Krajczar, Y. S. Lai, Y.-J. Lee, A. Levin, P. D. Luckey, A. C. Marini, C. Mcginn, C. Mironov, S. Narayanan, X. Niu, C. Paus, C. Roland, G. Roland, J. Salfeld-Nebgen, G. S. F. Stephans, K. Sumorok, K. Tatar, M. Varma, D. Velicanu, J. Veverka, J. Wang, T. W. Wang, B. Wyslouch, M. Yang, V. Zhukova, A. C. Benvenuti, R. M. Chatterjee, A. Evans, A. Finkel, A. Gude, P. Hansen, S. Kalafut, S. C. Kao, Y. Kubota, Z. Lesko, J. Mans, S. Nourbakhsh, N. Ruckstuhl, R. Rusack, N. Tambe, J. Turkewitz, J. G. Acosta, S. Oliveros, E. Avdeeva, R. Bartek, K. Bloom, S. Bose, D. R. Claes, A. Dominguez, C. Fangmeier, R. Gonzalez Suarez, R. Kamalieddin, D. Knowlton, I. Kravchenko, A. Malta Rodrigues, F. Meier, J. Monroy, J. E. Siado, G. R. Snow, B. Stieger, M. Alyari, J. Dolen, J. George, A. Godshalk, C. Harrington, I. Iashvili, J. Kaisen, A. Kharchilava, A. Kumar, A. Parker, S. Rappoccio, B. Roozbahani, G. Alverson, E. Barberis, D. Baumgartel, A. Hortiangtham, B. Knapp, A. Massironi, D. M. Morse, D. Nash, T. Orimoto, R. Teixeira De Lima, D. Trocino, R.-J. Wang, D. Wood, S. Bhattacharya, K. A. Hahn, A. Kubik, A. Kumar, J. F. Low, N. Mucia, N. Odell, B. Pollack, M. H. Schmitt, K. Sung, M. Trovato, M. Velasco, N. Dev, M. Hildreth, K. Hurtado Anampa, C. Jessop, D. J. Karmgard, N. Kellams, K. Lannon, N. Marinelli, F. Meng, C. Mueller, Y. Musienko, M. Planer, A. Reinsvold, R. Ruchti, G. Smith, S. Taroni, N. Valls, M. Wayne, M. Wolf, A. Woodard, J. Alimena, L. Antonelli, J. Brinson, B. Bylsma, L. S. Durkin, S. Flowers, B. Francis, A. Hart, C. Hill, R. Hughes, W. Ji, B. Liu, W. Luo, D. Puigh, B. L. Winer, H. W. Wulsin, S. Cooperstein, O. Driga, P. Elmer, J. Hardenbrook, P. Hebda, J. Luo, D. Marlow, T. Medvedeva, K. Mei, M. Mooney, J. Olsen, C. Palmer, P. Piroué, D. Stickland, C. Tully, A. Zuranski, S. Malik, A. Barker, V. E. Barnes, S. Folgueras, L. Gutay, M. K. Jha, M. Jones, A. W. Jung, K. Jung, D. H. Miller, N. Neumeister, B. C. Radburn-Smith, X. Shi, J. Sun, A. Svyatkovskiy, F. Wang, W. Xie, L. Xu, N. Parashar, J. Stupak, A. Adair, B. Akgun, Z. Chen, K. M. Ecklund, F. J. M. Geurts, M. Guilbaud, W. Li, B. Michlin, M. Northup, B. P. Padley, R. Redjimi, J. Roberts, J. Rorie, Z. Tu, J. Zabel, B. Betchart, A. Bodek, P. de Barbaro, R. Demina, Y. t. Duh, T. Ferbel, M. Galanti, A. Garcia-Bellido, J. Han, O. Hindrichs, A. Khukhunaishvili, K. H. Lo, P. Tan, M. Verzetti, J. P. Chou, E. Contreras-Campana, Y. Gershtein, T. A. Gómez Espinosa, E. Halkiadakis, M. Heindl, D. Hidas, E. Hughes, S. Kaplan, R. Kunnawalkam Elayavalli, S. Kyriacou, A. Lath, K. Nash, H. Saka, S. Salur, S. Schnetzer, D. Sheffield, S. Somalwar, R. Stone, S. Thomas, P. Thomassen, M. Walker, M. Foerster, J. Heideman, G. Riley, K. Rose, S. Spanier, K. Thapa, O. Bouhali, A. Celik, M. Dalchenko, M. De Mattia, A. Delgado, S. Dildick, R. Eusebi, J. Gilmore, T. Huang, E. Juska, T. Kamon, R. Mueller, Y. Pakhotin, R. Patel, A. Perloff, L. Perniè, D. Rathjens, A. Rose, A. Safonov, A. Tatarinov, K. A. Ulmer, N. Akchurin, C. Cowden, J. Damgov, C. Dragoiu, P. R. Dudero, J. Faulkner, S. Kunori, K. Lamichhane, S. W. Lee, T. Libeiro, S. Undleeb, I. Volobouev, Z. Wang, A. G. Delannoy, S. Greene, A. Gurrola, R. Janjam, W. Johns, C. Maguire, A. Melo, H. Ni, P. Sheldon, S. Tuo, J. Velkovska, Q. Xu, M. W. Arenton, P. Barria, B. Cox, J. Goodell, R. Hirosky, A. Ledovskoy, H. Li, C. Neu, T. Sinthuprasith, X. Sun, Y. Wang, E. Wolfe, F. Xia, C. Clarke, R. Harr, P. E. Karchin, P. Lamichhane, J. Sturdy, D. A. Belknap, S. Dasu, L. Dodd, S. Duric, B. Gomber, M. Grothe, M. Herndon, A. Hervé, P. Klabbers, A. Lanaro, A. Levine, K. Long, R. Loveless, I. Ojalvo, T. Perry, G. A. Pierro, G. Polese, T. Ruggles, A. Savin, A. Sharma, N. Smith, W. H. Smith, D. Taylor, N. Woods, CMS Collaboration

**Affiliations:** 1 Yerevan Physics Institute, Yerevan, Armenia; 2 Institut für Hochenergiephysik der OeAW, Wien, Austria; 3 National Centre for Particle and High Energy Physics, Minsk, Belarus; 4 Universiteit Antwerpen, Antwerp, Belgium; 5 Vrije Universiteit Brussel, Brussels, Belgium; 6 Université Libre de Bruxelles, Brussels, Belgium; 7 Ghent University, Ghent, Belgium; 8 Université Catholique de Louvain, Louvain-la-Neuve, Belgium; 9 Université de Mons, Mons, Belgium; 10 Centro Brasileiro de Pesquisas Fisicas, Rio de Janeiro, Brazil; 11 Universidade do Estado do Rio de Janeiro, Rio de Janeiro, Brazil; 12 Universidade Estadual Paulista, Universidade Federal do ABC, São Paulo, Brazil; 13 Institute for Nuclear Research and Nuclear Energy, Sofia, Bulgaria; 14 University of Sofia, Sofia, Bulgaria; 15 Beihang University, Beijing, China; 16 Institute of High Energy Physics, Beijing, China; 17 State Key Laboratory of Nuclear Physics and Technology, Peking University, Beijing, China; 18 Universidad de Los Andes, Bogotá, Colombia; 19 Faculty of Electrical Engineering, Mechanical Engineering and Naval Architecture, University of Split, Split, Croatia; 20 Faculty of Science, University of Split, Split, Croatia; 21 Institute Rudjer Boskovic, Zagreb, Croatia; 22 University of Cyprus, Nicosia, Cyprus; 23 Charles University, Prague, Czech Republic; 24 Universidad San Francisco de Quito, Quito, Ecuador; 25 Academy of Scientific Research and Technology of the Arab Republic of Egypt, Egyptian Network of High Energy Physics, Cairo, Egypt; 26 National Institute of Chemical Physics and Biophysics, Tallinn, Estonia; 27 Department of Physics, University of Helsinki, Helsinki, Finland; 28 Helsinki Institute of Physics, Helsinki, Finland; 29 Lappeenranta University of Technology, Lappeenranta, Finland; 30 IRFU, CEA, Université Paris-Saclay, Gif-sur-Yvette, France; 31 Laboratoire Leprince-Ringuet, Ecole Polytechnique, IN2P3-CNRS, Palaiseau, France; 32 Institut Pluridisciplinaire Hubert Curien, Université de Strasbourg, Université de Haute Alsace Mulhouse, CNRS/IN2P3, Strasbourg, France; 33 Centre de Calcul de l’Institut National de Physique Nucleaire et de Physique des Particules, CNRS/IN2P3, Villeurbanne, France; 34 Université de Lyon, Université Claude Bernard Lyon 1, CNRS-IN2P3, Institut de Physique Nucléaire de Lyon, Villeurbanne, France; 35 Georgian Technical University, Tbilisi, Georgia; 36 Tbilisi State University, Tbilisi, Georgia; 37 RWTH Aachen University, I. Physikalisches Institut, Aachen, Germany; 38 RWTH Aachen University, III. Physikalisches Institut A, Aachen, Germany; 39 RWTH Aachen University, III. Physikalisches Institut B, Aachen, Germany; 40 Deutsches Elektronen-Synchrotron, Hamburg, Germany; 41 University of Hamburg, Hamburg, Germany; 42 Institut für Experimentelle Kernphysik, Karlsruhe, Germany; 43 Institute of Nuclear and Particle Physics (INPP), NCSR Demokritos, Aghia Paraskevi, Greece; 44 National and Kapodistrian University of Athens, Athens, Greece; 45 University of Ioánnina, Ioannina, Greece; 46 MTA-ELTE Lendület CMS Particle and Nuclear Physics Group, Eötvös Loránd University, Budapest, Hungary; 47 Wigner Research Centre for Physics, Budapest, Hungary; 48 Institute of Nuclear Research ATOMKI, Debrecen, Hungary; 49 University of Debrecen, Debrecen, Hungary; 50 National Institute of Science Education and Research, Bhubaneswar, India; 51 Panjab University, Chandigarh, India; 52 University of Delhi, Delhi, India; 53 Saha Institute of Nuclear Physics, Kolkata, India; 54 Indian Institute of Technology Madras, Madras, India; 55 Bhabha Atomic Research Centre, Mumbai, India; 56 Tata Institute of Fundamental Research-A, Mumbai, India; 57 Tata Institute of Fundamental Research-B, Mumbai, India; 58 Indian Institute of Science Education and Research (IISER), Pune, India; 59 Institute for Research in Fundamental Sciences (IPM), Tehran, Iran; 60 University College Dublin, Dublin, Ireland; 61 INFN Sezione di Bari, Università di Bari, Politecnico di Bari, Bari, Italy; 62 INFN Sezione di Bologna, Università di Bologna, Bologna, Italy; 63 INFN Sezione di Catania, Università di Catania, Catania, Italy; 64 INFN Sezione di Firenze, Università di Firenze, Florence, Italy; 65 INFN Laboratori Nazionali di Frascati, Frascati, Italy; 66 INFN Sezione di Genova, Università di Genova, Genoa, Italy; 67 INFN Sezione di Milano-Bicocca, Università di Milano-Bicocca, Milan, Italy; 68 INFN Sezione di Napoli, Università di Napoli ’Federico II’ Naples, Italy, Università della Basilicata, Potenza, Italy, Università G. Marconi, Rome, Italy; 69 INFN Sezione di Padova, Università di Padova, Padua, Italy, Università di Trento, Trento, Italy; 70 INFN Sezione di Pavia, Università di Pavia, Pavia, Italy; 71 INFN Sezione di Perugia, Università di Perugia, Perugia, Italy; 72 INFN Sezione di Pisa, Università di Pisa, Scuola Normale Superiore di Pisa, Pisa, Italy; 73 INFN Sezione di Roma, Università di Roma, Rome, Italy; 74 INFN Sezione di Torino, Università di Torino, Turin, Italy, Università del Piemonte Orientale, Novara, Italy; 75 INFN Sezione di Trieste, Università di Trieste, Trieste, Italy; 76 Kyungpook National University, Daegu, Korea; 77 Chonbuk National University, Jeonju, Korea; 78 Hanyang University, Seoul, Korea; 79 Korea University, Seoul, Korea; 80 Seoul National University, Seoul, Korea; 81 University of Seoul, Seoul, Korea; 82 Sungkyunkwan University, Suwon, Korea; 83 Vilnius University, Vilnius, Lithuania; 84 National Centre for Particle Physics, Universiti Malaya, Kuala Lumpur, Malaysia; 85 Centro de Investigacion y de Estudios Avanzados del IPN, Mexico City, Mexico; 86 Universidad Iberoamericana, Mexico City, Mexico; 87 Benemerita Universidad Autonoma de Puebla, Puebla, Mexico; 88 Universidad Autónoma de San Luis Potosí, San Luis Potosí, Mexico; 89 University of Auckland, Auckland, New Zealand; 90 University of Canterbury, Christchurch, New Zealand; 91 National Centre for Physics, Quaid-I-Azam University, Islamabad, Pakistan; 92 National Centre for Nuclear Research, Swierk, Poland; 93 Institute of Experimental Physics, Faculty of Physics, University of Warsaw, Warsaw, Poland; 94 Laboratório de Instrumentação e Física Experimental de Partículas, Lisbon, Portugal; 95 Joint Institute for Nuclear Research, Dubna, Russia; 96 Petersburg Nuclear Physics Institute, Gatchina (St. Petersburg), Russia; 97 Institute for Nuclear Research, Moscow, Russia; 98 Institute for Theoretical and Experimental Physics, Moscow, Russia; 99 Moscow Institute of Physics and Technology, Moscow, Russia; 100 National Research Nuclear University ‘Moscow Engineering Physics Institute’ (MEPhI), Moscow, Russia; 101 P.N. Lebedev Physical Institute, Moscow, Russia; 102 Skobeltsyn Institute of Nuclear Physics, Lomonosov Moscow State University, Moscow, Russia; 103 Novosibirsk State University (NSU), Novosibirsk, Russia; 104 State Research Center of Russian Federation, Institute for High Energy Physics, Protvino, Russia; 105 Faculty of Physics and Vinca Institute of Nuclear Sciences, University of Belgrade, Belgrade, Serbia; 106 Centro de Investigaciones Energéticas Medioambientales y Tecnológicas (CIEMAT), Madrid, Spain; 107 Universidad Autónoma de Madrid, Madrid, Spain; 108 Universidad de Oviedo, Oviedo, Spain; 109 Instituto de Física de Cantabria (IFCA), CSIC-Universidad de Cantabria, Santander, Spain; 110 CERN, European Organization for Nuclear Research, Geneva, Switzerland; 111 Paul Scherrer Institut, Villigen, Switzerland; 112 Institute for Particle Physics, ETH Zurich, Zurich, Switzerland; 113 Universität Zürich, Zurich, Switzerland; 114 National Central University, Chung-Li, Taiwan; 115 National Taiwan University (NTU), Taipei, Taiwan; 116 Chulalongkorn University, Faculty of Science, Department of Physics, Bangkok, Thailand; 117 Cukurova University, Adana, Turkey; 118 Middle East Technical University, Physics Department, Ankara, Turkey; 119 Bogazici University, Istanbul, Turkey; 120 Istanbul Technical University, Istanbul, Turkey; 121 Institute for Scintillation Materials of National Academy of Science of Ukraine, Kharkov, Ukraine; 122 National Scientific Center, Kharkov Institute of Physics and Technology, Kharkov, Ukraine; 123 University of Bristol, Bristol, UK; 124 Rutherford Appleton Laboratory, Didcot, UK; 125 Imperial College, London, UK; 126 Brunel University, Uxbridge, UK; 127 Baylor University, Waco, USA; 128 The University of Alabama, Tuscaloosa, USA; 129 Boston University, Boston, USA; 130 Brown University, Providence, USA; 131 University of California, Davis, Davis, USA; 132 University of California, Los Angeles, USA; 133 University of California, Riverside, Riverside, USA; 134 University of California, San Diego, La Jolla, USA; 135 Department of Physics, University of California, Santa Barbara, Santa Barbara, USA; 136 California Institute of Technology, Pasadena, USA; 137 Carnegie Mellon University, Pittsburgh, USA; 138 University of Colorado Boulder, Boulder, USA; 139 Cornell University, Ithaca, USA; 140 Fairfield University, Fairfield, USA; 141 Fermi National Accelerator Laboratory, Batavia, USA; 142 University of Florida, Gainesville, USA; 143 Florida International University, Miami, USA; 144 Florida State University, Tallahassee, USA; 145 Florida Institute of Technology, Melbourne, USA; 146 University of Illinois at Chicago (UIC), Chicago, USA; 147 The University of Iowa, Iowa City, USA; 148 Johns Hopkins University, Baltimore, USA; 149 The University of Kansas, Lawrence, USA; 150 Kansas State University, Manhattan, USA; 151 Lawrence Livermore National Laboratory, Livermore, USA; 152 University of Maryland, College Park, USA; 153 Massachusetts Institute of Technology, Cambridge, USA; 154 University of Minnesota, Minneapolis, USA; 155 University of Mississippi, Oxford, USA; 156 University of Nebraska-Lincoln, Lincoln, USA; 157 State University of New York at Buffalo, Buffalo, USA; 158 Northeastern University, Boston, USA; 159 Northwestern University, Evanston, USA; 160 University of Notre Dame, Notre Dame, USA; 161 The Ohio State University, Columbus, USA; 162 Princeton University, Princeton, USA; 163 University of Puerto Rico, Mayaguez, USA; 164 Purdue University, West Lafayette, USA; 165 Purdue University Calumet, Hammond, USA; 166 Rice University, Houston, USA; 167 University of Rochester, Rochester, USA; 168 Rutgers, The State University of New Jersey, Piscataway, USA; 169 University of Tennessee, Knoxville, USA; 170 Texas A&M University, College Station, USA; 171 Texas Tech University, Lubbock, USA; 172 Vanderbilt University, Nashville, USA; 173 University of Virginia, Charlottesville, USA; 174 Wayne State University, Detroit, USA; 175 University of Wisconsin-Madison, Madison, WI USA; 176 CERN, 1211 Geneva 23, Switzerland

## Abstract

The WZ production cross section is measured by the CMS experiment at the CERN LHC in proton–proton collision data samples corresponding to integrated luminosities of 4.9$\,\text{fb}^{-1}$ collected at $\sqrt{s} = 7\,\text{TeV}$, and 19.6$\,\text{fb}^{-1}$ at $\sqrt{s} = 8\,\text{TeV}$. The measurements are performed using the fully-leptonic WZ decay modes with electrons and muons in the final state. The measured cross sections for $71< m_{\mathrm{Z}} < 111\,\text{GeV}$ are $\sigma ({\mathrm{p}\mathrm{p}}\rightarrow {\mathrm{W}\mathrm{Z}};~\sqrt{s} = 7\,\text{TeV}) = 20.14 \pm 1.32\,\text{(stat)} \pm 0.38\,\text{(theo)} \pm 1.06\,\text{(exp)} \pm 0.44\,\text{(lumi)}$ $\,\text{pb}$ and $\sigma ({\mathrm{p}\mathrm{p}}\rightarrow {\mathrm{W}\mathrm{Z}};~\sqrt{s} = 8\,\text{TeV}) = 24.09 \pm 0.87\,\text{(stat)} \pm 0.80\,\text{(theo)} \pm 1.40\,\text{(exp)} \pm 0.63\,\text{(lumi)}$ $\,\text{pb}$. Differential cross sections with respect to the $\mathrm{Z}$ boson $p_{\mathrm{T}}$, the leading jet $p_{\mathrm{T}}$, and the number of jets are obtained using the $\sqrt{s} = 8\,\text{TeV}$ data. The results are consistent with standard model predictions and constraints on anomalous triple gauge couplings are obtained.

## Introduction

The measurement of the production of electroweak heavy vector boson pairs (diboson production) in proton–proton collisions represents an important test of the standard model (SM) description of electroweak and strong interactions at the $\,\text{TeV}$ scale. Diboson production is sensitive to the self-interactions between electroweak gauge bosons as predicted by the $\mathcal {SU}(2)_{L} \times \mathcal {U}(1)_{Y}$ gauge structure of electroweak interactions. Triple and quartic gauge couplings (TGCs and QGCs) can be affected by new physics phenomena involving new particles at higher energy scales. The $\mathrm{W}\mathrm{Z}$ cross section measured in this paper is sensitive to WWZ couplings, which are non-zero in the SM. WZ production also represents an important background in several searches for physics beyond the SM, such as the search for the SM Higgs boson [1], searches for new resonances [2, 3], or supersymmetry [4–7].

**Fig. 1:**
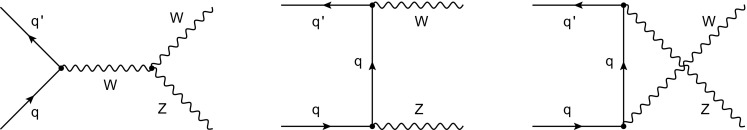
Leading-order Feynman diagrams for WZ production in proton–proton collisions. The three diagrams represent contributions from (*left*) *s*-channel through TGC, (*middle*) *t*-channel, and (*right*) *u*-channel

We present a study of WZ production in proton–proton collisions based on data recorded by the CMS detector at the CERN LHC in 2011 and 2012, corresponding to integrated luminosities of 4.9$\,\text{fb}^{-1}$ collected at $\sqrt{s}=7\,\text{TeV}$, and 19.6$\,\text{fb}^{-1}$ collected at $\sqrt{s}=8\,\text{TeV}$. The measurements use purely leptonic final states in which the Z boson decays into a pair of electrons or muons, and the W boson decays into a neutrino and an electron or a muon. At leading order (LO) within the SM, WZ production in proton–proton collisions occurs through quark–antiquark interactions in the *s*-, *t*-, and *u*-channels, as illustrated by the Feynman diagrams shown in Fig. 1. Among them, only the *s*-channel includes a TGC vertex. Our measured final states also include contributions from diagrams where the $\mathrm{Z}$ boson is replaced with a virtual photon ($\gamma ^*$) and thus include $\mathrm{W}\gamma ^*$ production. We refer to the final states as $\mathrm{W}\mathrm{Z}$ production because the $\mathrm{Z}$ contribution is dominant for the phase space of this measurement. Hadron collider WZ production has been previously observed at both the Tevatron [8, 9] and the LHC [10–15].

We first describe measurements of the inclusive WZ production cross section at both centre-of-mass energies. The measurements are restricted to the phase space in which the invariant mass of the two leptons from the Z boson decay lies within 20$\,\text{GeV}$ of the nominal Z boson mass [16]. Using the larger integrated luminosity collected at $\sqrt{s}=8\,\text{TeV}$, we also present measurements of the differential cross section as a function of the Z boson transverse momentum $p_{\mathrm{T}}$, the number of jets produced in association with the $\mathrm{W}\mathrm{Z}$ pair, and the $p_{\mathrm{T}}$ of the leading associated jet. The measurements involving jets are especially useful for probing the contribution of higher-order QCD processes to the cross section.

Finally, we present a search for anomalous WWZ couplings based on a measurement of the $p_{\mathrm{T}}$ spectrum of the Z boson. The search is formulated both in the framework of anomalous couplings and in an effective field theory approach.

## The CMS detector

The central feature of the CMS apparatus is a superconducting solenoid of 6$\,\text{m}$ internal diameter, providing a magnetic field of 3.8$\,\text{T}$. Within the solenoid volume are a silicon pixel and strip tracker, a lead tungstate crystal electromagnetic calorimeter (ECAL), and a brass and scintillator hadron calorimeter (HCAL), each composed of a barrel and two endcap sections. Muons are measured in gas-ionization detectors embedded in the steel flux-return yoke outside the solenoid with detection planes made using three technologies: drift tubes, cathode strip chambers, and resistive-plate chambers. Extensive forward calorimetry complements the coverage provided by the barrel and endcap detectors. The silicon tracker measures charged particles within the pseudorapidity range $|\eta |< 2.50$. The ECAL provides coverage in $| \eta |< 1.48$ in a barrel region and $1.48<|\eta | < 3.00$ in two endcap regions. Muons are measured in the range $|\eta |< 2.40$.

A more detailed description of the CMS detector, together with a definition of the coordinate system used and the relevant kinematic variables, can be found in Ref. [17].

## Simulated samples

Several Monte Carlo (MC) event generators are used to simulate signal and background processes. The W($\mathrm{Z}/\gamma ^*$) signal for $m_{\mathrm{Z}/\gamma ^*} > 12 \,\text{GeV}$ is generated at LO with MadGraph 5.1 [18] with up to two additional partons at matrix element level. The $\mathrm{t}\overline{\mathrm{t}}$, tW, and $\mathrm{q} \overline{\mathrm{q}} \rightarrow \mathrm{Z} \mathrm{Z}$ processes are generated at next-to-leading order (NLO) with powheg 2.0 [19–21]. The ${\mathrm{g} \mathrm{g} \rightarrow \mathrm{Z} \mathrm{Z}}$ process is simulated at leading order (one loop) with gg2zz [22]. Other background processes are generated at LO with MadGraph and include $\mathrm{Z}$ +jets, $\mathrm{W}\gamma ^*$ (with $m_{\gamma *}<12\,\text{GeV}$), $\mathrm{Z} \gamma$ as well as processes with at least three bosons in the decay chain comprised of $\mathrm{W}\mathrm{Z} \mathrm{Z}$, $\mathrm{Z} \mathrm{Z} \mathrm{Z}$, $\mathrm{W}\mathrm{W}\mathrm{Z}$, $\mathrm{W}\mathrm{W}\mathrm{W}$, $\mathrm{t}\overline{\mathrm{t}} \mathrm{W}$, $\mathrm{t}\overline{\mathrm{t}} \mathrm{Z}$, $\mathrm{t}\overline{\mathrm{t}} \mathrm{W}\mathrm{W}$, $\mathrm{t}\overline{\mathrm{t}} \gamma$ and $\mathrm{W}\mathrm{W}\gamma$, collectively referred to as VVV. For the modeling of anomalous triple gauge couplings (aTGCs), the NLO mcfm 6.3 [23] Monte Carlo program is used to compute weights that are applied to the WZ signal sample generated with MadGraph. In all samples, the parton-level events are interfaced with pythia 6.426 [24] to describe parton showering, hadronization, fragmentation, and the underlying event with the Z2* tune [25]. For LO generators, the default set of parton distribution functions (PDFs) used is CTEQ6L1 [26], while NLO CT10 [27] is used with NLO generators. For all processes, the detector response is simulated with a detailed description of the CMS detector, based on the Geant4  package [28]. The event reconstruction is performed with the same algorithms as are used for data. The simulated samples include additional interactions per bunch crossing (pileup). Simulated events are weighted so the pileup distribution in the simulation matches the one observed in data.

## Event reconstruction and object identification

The measurement of the $\mathrm{W}\mathrm{Z} \rightarrow \ell \nu \ell '\ell '$ decay, where $\ell ,\ell '=\mathrm{e}$ or $\mu$, relies on the effective identification of electrons and muons, and an accurate measurement of missing transverse momentum. The lepton selection requirements used in this measurement are the same as those used in the Higgs boson $\mathrm{H} \rightarrow {\mathrm{W}\mathrm{W}} \rightarrow \ell \ell ^\prime \nu \nu$ measurement [1]. The kinematic properties of the final-state leptons in those two processes are very similar and the two measurements are affected by similar sources of lepton backgrounds.

Events are required to be accepted by one of the following double-lepton triggers: two electrons or two muons with transverse momentum thresholds of 17$\,\text{GeV}$ for the leading lepton, and 8$\,\text{GeV}$ for the trailing one. For the 8$\,\text{TeV}$ data sample, events are also accepted when an electron-muon pair satisfies the same momentum criteria.

A particle-flow (PF) algorithm [29, 30] is used to reconstruct and identify each individual particle with an optimized combination of information from the various elements of the CMS detector: clusters of energy deposits measured by the calorimeters, and charged-particle tracks identified in the central tracking system and the muon detectors.

Electrons are reconstructed by combining information from the ECAL and tracker [31]. Their identification relies on a multivariate regression technique that combines observables sensitive to the amount of bremsstrahlung along the electron trajectory, the geometrical and momentum matching between the electron trajectory in the tracker and the energy deposit in the calorimeter, as well as the shower shape. Muons are reconstructed using information from both the tracker and the muon spectrometer [32]. They must satisfy requirements on the number of hits in the layers of the tracker and in the muon spectrometer, and on the quality of the full track fit. All lepton candidates are required to be consistent with the primary vertex of the event, which is chosen as the vertex with the highest $\sum p_{\mathrm{T}} ^2$ of its associated tracks. This criterion provides the correct assignment for the primary vertex in more than 99% of both signal and background events for the pileup distribution observed in data. Both electrons and muons are required to have $p_{\mathrm{T}} >10\,\text{GeV}$. Electrons (muons) must satisfy $|\eta |<2.5$ (2.4).

Charged leptons from $\mathrm{W}$ and $\mathrm{Z}$ boson decays are mostly isolated from other final-state particles in the event. Consequently, the selected leptons are required to be isolated from other activity in the event to reduce the backgrounds from hadrons that are misidentified as leptons or from leptons produced in hadron decays when they occur inside or near hadronic jets. The separation between two reconstructed objects in the detector is measured with the variable $\Delta R = \sqrt{ (\Delta \eta )^2 + (\Delta \phi )^2}$, where $\phi$ is the azimuthal angle. To measure the lepton isolation, we consider a $\Delta R=0.3$ cone around the lepton candidate track direction at the event vertex. An isolation variable is then built as the scalar $p_{\mathrm{T}}$ sum of all PF objects consistent with the chosen primary vertex, and contained within the cone. The contribution from the lepton candidate itself is excluded. For both electrons and muons a correction is applied to account for the energy contribution in the isolation cone due to pileup. In the case of electrons, the average energy density in the isolation cone due to pileup is determined event-by-event and is used to correct the isolation variable [33]. For muons, the pileup contribution from neutral particles to the isolation is estimated using charged particles associated with pileup interactions. This isolation variable is required to be smaller than about 10% of the candidate lepton $p_{\mathrm{T}}$. The exact threshold value depends on the lepton flavour and detector region, and also on the data taking period: for 7$\,\text{TeV}$ data, it is 13% (9%) for electrons measured in the ECAL barrel (endcaps) and 12% for muons, while for 8$\,\text{TeV}$ data it is 15% for all electrons. For muons, a modified strategy has been used for 8$\,\text{TeV}$ data to account for the higher pileup conditions in order to reduce the dependence of this variable on the number of pileup interactions. It uses a multivariate algorithm based on the $p_{\mathrm{T}}$ sums of particles around the lepton candidates built for $\Delta R$ cones of different sizes [1].

The lepton reconstruction and selection efficiencies and associated uncertainties are determined using a tag-and-probe method with $\mathrm{Z} \rightarrow \ell \ell$ events [34] chosen using the same criteria in data and simulation in several ($p_{\mathrm{T}}$,$\eta$) bins. Ratios of efficiencies from data and simulation are calculated for each bin. To account for differences between data and simulation, the simulated samples are reweighted by these ratios for each selected lepton in the event. The total uncertainty for the lepton efficiencies, including effects from trigger, reconstruction, and selection amounts to roughly 2% per lepton. The lepton selection criteria in the 7 and 8$\,\text{TeV}$ samples are chosen to maintain a stable efficiency throughout each data sample.

Jets are reconstructed from PF objects using the anti-$k_{\mathrm{T}}$ clustering algorithm [35, 36] with a size parameter *R* of 0.5. The energy of photons is obtained from the ECAL measurement. The energy of electrons is determined from a combination of the electron momentum at the primary interaction vertex as determined by the tracker, the energy of the corresponding ECAL cluster, and the energy sum of all bremsstrahlung photons spatially compatible with origination from the electron track. The energy of muons is obtained from the curvature of the corresponding track. The energy of charged hadrons is determined from a combination of their momentum measured in the tracker and the matching ECAL and HCAL energy deposits, corrected for the response function of the calorimeters to hadronic showers. Finally, the energy of neutral hadrons is obtained from the corresponding corrected ECAL and HCAL energy. The jet momentum is determined as the vector sum of all particle momenta in the jet. A correction is applied to jet energies to take into account the contribution from pileup. Jet energy corrections are derived from the simulation, and are confirmed with in situ measurements with the energy balance of dijet and photon + jet events [37]. The jet energy resolution amounts typically to 15% at 10$\,\text{GeV}$, 8% at 100$\,\text{GeV}$, and 4% at 1$\,\text{TeV}$. Additional selection criteria are applied to each event to remove spurious jet-like features originating from isolated noise patterns in certain HCAL regions.

The missing transverse momentum vector ${\vec p}_{\mathrm{T}}^{\text{miss}}$ is defined as the negative vector sum of the transverse momenta of all reconstructed particles in an event. Its magnitude is referred to as $E_{\mathrm{T}}^{\text{miss}}$.

## Event selection and background estimates

We select $\mathrm{W}\mathrm{Z}\rightarrow \ell \nu \ell '\ell '$ decays with $\mathrm{W}\rightarrow \ell \nu$ and $\mathrm{Z}\rightarrow \ell '\ell '$, where $\ell$ and $\ell '$ are electrons or muons. These decays are characterized by a pair of same-flavour, opposite-charge, isolated leptons with an invariant mass consistent with a Z boson, together with a third isolated lepton and a significant amount of missing transverse energy $E_{\mathrm{T}}^{\text{miss}}$ associated with the escaping neutrino. We consider four different signatures corresponding to the flavour of the leptons in the final state: $\mathrm{e}\mathrm{e}\mathrm{e}$, $\mathrm{e}\mathrm{e}\mu$, $\mathrm{e}\mu \mu$ and $\mu \mu \mu$.

The four final states are treated independently for the cross section measurements and for the search for anomalous couplings, and are combined only at the level of the final results. Unless explicitly stated otherwise, identical selection criteria are applied to the 7 and 8$\,\text{TeV}$ samples.

Candidate events are triggered by requiring the presence of two electrons or two muons. In the 8$\,\text{TeV}$ sample, events triggered by the presence of an electron and a muon are also accepted. The trigger efficiency for signal-like events that pass the event selection is measured to be larger than 99%. The candidate events are required to contain exactly three leptons matching all selection criteria. In the 8$\,\text{TeV}$ analysis, the invariant mass of the three leptons is required to be larger than 100$\,\text{GeV}$. The $\mathrm{Z}$ boson candidates are built from two oppositely charged, same-flavour, isolated leptons. The leading lepton is required to have $p_{\mathrm{T}} > 20\,\text{GeV}$. The Z boson candidate invariant mass should lie within 20$\,\text{GeV}$ of the nominal Z boson mass: $71< m_{\ell \ell } < 111\,\text{GeV}$. If two matching pairs are found, the Z boson candidate with the mass closest to the nominal $\mathrm{Z}$ boson mass is selected. The remaining lepton is associated with the W boson and is required to have $p_{\mathrm{T}} > 20\,\text{GeV}$ and to be separated from both leptons in the $\mathrm{Z}$ boson decay by $\Delta R > 0.1$. Finally, to account for the escaping neutrino, $E_{\mathrm{T}}^{\text{miss}}$ is required to be larger than 30$\,\text{GeV}$.

Background sources with three reconstructed leptons include events with prompt leptons produced at the primary vertex or leptons from displaced vertices, as well as jets.

The background contribution from nonprompt leptons, dominated by $\mathrm{t}\overline{\mathrm{t}}$ and Z+jets events in which one of the three reconstructed leptons is misidentified, is estimated using a procedure similar to Ref. [38]. In this procedure, the amount of background in the signal region is estimated using the yields observed in several mutually exclusive samples containing events that did not satisfy some of the lepton selection requirements. The method uses the distinction between a loose and a tight lepton selection. The tight selection is identical to the one used in the final selection, while some of the lepton identification requirements used in the final selection are relaxed in the loose selection. The procedure starts from a sample, called the loose sample, with three leptons passing loose identification criteria and otherwise satisfying all other requirements of the WZ selection. This sample receives contributions from events with three prompt (p) leptons, two prompt leptons and one nonprompt (n) lepton, one prompt lepton and two nonprompt leptons, and three nonprompt leptons. The event yield of the loose sample $N_{\mathrm{LLL}}$ can thus be expressed as,

1 $$\begin{aligned} N_{\mathrm{LLL}} = n_{\mathrm{ppp}} + n_{\mathrm{ppn}} + n_{\mathrm{pnp}} + n_{\mathrm{npp}} + n_{\mathrm{nnp}} + n_{\mathrm{npn}} + n_{\mathrm{pnn}} + n_{\mathrm{nnn}}. \end{aligned}$$

In this expression, the first, second and third indices refer to the leading and subleading leptons from the Z boson decay and to the lepton from the W boson decay, respectively. The loose sample can be divided into subsamples depending on whether each of the three leptons passes or fails the tight selection. The number of events in each subsample is labeled $N_{ijk}$ with $i,j,k = \mathrm {T,F}$ where T and F stand for leptons passing or failing the tight selection, respectively. The yield in each of these subsamples can be expressed as a linear combination of the unknown yields $n_{\alpha \beta \gamma }$ ($\alpha ,\beta ,\gamma \, \in \{ \mathrm {p,n} \}$),

2 $$\begin{aligned} N_{ijk} = \sum _{\alpha ,\beta ,\gamma \, \in \{ \mathrm {p,n} \}} C^{ijk}_{\alpha \beta \gamma } n_{\alpha \beta \gamma }, \quad i,j,k=\mathrm {T,F}, \end{aligned}$$

where the coefficients $C^{ijk}_{\alpha \beta \gamma }$ depend on the efficiencies $\epsilon _{\mathrm{p}}$ and $\epsilon _{\mathrm{n}}$, which stand for the probabilities of prompt and nonprompt leptons, respectively, to pass the tight lepton selection provided they have passed the loose selection. For example, starting from Eq. (1), the number of events with all three leptons passing the tight selection $N_{\mathrm{TTT}}$ can be written as

3 $$\begin{aligned} N_{\mathrm{TTT}}= & {} n_{\mathrm{ppp}}\epsilon _{\mathrm{p}_1}\epsilon _{\mathrm{p}_2} \epsilon _{\mathrm{p}_3} + n_{\mathrm{ppn}}\epsilon _{\mathrm{p}_1}\epsilon _{\mathrm{p}_2} \epsilon _{\mathrm{n}_3} + n_{\mathrm{pnp}}\epsilon _{\mathrm{p}_1}\epsilon _{\mathrm{n}_2} \epsilon _{\mathrm{p}_3} \nonumber \\&+ n_{\mathrm{npp}}\epsilon _{\mathrm{n}_1}\epsilon _{\mathrm{p}_2} \epsilon _{\mathrm{p}_3} + n_{\mathrm{nnp}}\epsilon _{\mathrm{n}_1}\epsilon _{\mathrm{n}_2} \epsilon _{\mathrm{p}_3} + n_{\mathrm{npn}}\epsilon _{\mathrm{n}_1}\epsilon _{\mathrm{p}_2} \epsilon _{\mathrm{n}_3} \nonumber \\&+ n_{\mathrm{pnn}}\epsilon _{\mathrm{p}_1}\epsilon _{\mathrm{n}_2} \epsilon _{\mathrm{n}_3} + n_{\mathrm{nnn}}\epsilon _{\mathrm{n}_1}\epsilon _{\mathrm{n}_2} \epsilon _{\mathrm{n}_3}. \end{aligned}$$

The goal is to determine the number of events with three prompt leptons in the TTT sample, corresponding exactly to the selection used to perform the measurement. This yield is $n_{\mathrm{ppp}}\epsilon _{\mathrm{p}_1} \epsilon _{\mathrm{p}_2} \epsilon _{\mathrm{p}_3}$. The number of events with three prompt leptons in the loose sample, $n_{\mathrm{ppp}}$, is obtained by solving the set of linear Eq. (2).

**Table 1:** Expected and observed event yields at $\sqrt{s} = 7$ and 8$\,\text{TeV}$. The contributions from $\mathrm{t}\overline{\mathrm{t}}$, Z+jets, and other processes with nonprompt leptons have been determined from data control samples, as described in the text. Backgrounds with at least three bosons in the decay chain comprised of WZZ, $\mathrm{Z} \mathrm{Z} \mathrm{Z}$, WWZ, WWW, $\mathrm{t}\overline{\mathrm{t}} \mathrm{W}$, $\mathrm{t}\overline{\mathrm{t}} \mathrm{Z}$, $\mathrm{t}\overline{\mathrm{t}} \mathrm{W}\mathrm{W}$, $\mathrm{t}\overline{\mathrm{t}} \gamma$ and $\mathrm{W}\mathrm{W}\gamma$ events, are referred to as $\mathrm{V} \mathrm{V} \mathrm{V}$. Combined statistical and systematic uncertainties are shown, except for the WZ signal where only statistical uncertainties are shown

Sample	$\mathrm{e}\mathrm{e}\mathrm{e}$	$\mathrm{e}\mathrm{e}\mu$	$\mu \mu \mathrm{e}$	$\mu \mu \mu$	Total
$\sqrt{s} = 7\,\text{TeV}$; ${\mathcal {L}} = 4.9\,\text{fb}^{-1}$					
Nonprompt leptons	2.2 ± 2.1	1.5$^{+4.8}_{-1.5}$	2.4$^{+5.1}_{-2.4}$	1.8$^{+7.5}_{-1.8}$	7.9$^{+13.0}_{-5.0}$
ZZ	2.0 ± 0.3	3.5 ± 0.5	2.7 ± 0.4	5.1 ± 0.7	13.3 ± 1.9
Z$\gamma$	0	0	0.5 ± 0.5	0	0.5 ± 0.5
VVV	1.6 ± 0.8	2.0 ± 1.0	2.4 ± 1.2	3.0 ± 1.5	9.0 ± 4.5
Total background ($N_{\text{bkg}}$)	3.8 ± 2.3	6.0 ± $^{+4.9}_{-1.9}$	8.0$^{+5.1}_{-2.4}$	9.9$^{+7.7}_{-2.4}$	30.7$^{+13.9}_{-7.0}$
WZ	44.7 ± 0.5	49.8 ± 0.5	56.0 ± 0.5	73.8 ± 0.6	224.3 ± 1.1
Total expected	50.5 ± 2.3	56.8$^{+5.0}_{-1.9}$	64.0$^{+5.3}_{-2.8}$	83.7$^{+7.7}_{-2.5}$	255$^{+14.0}_{-7.0}$
Data ($N_{\text{obs}}$)	64	62	70	97	293
$\sqrt{s} = 8\,\text{TeV}$; ${\mathcal {L}} = 19.6\,\text{fb}^{-1}$					
Nonprompt leptons	18.4 ± 12.7	32.0 ± 21.0	54.4 ± 33.0	62.4 ± 37.7	167.1 ± 55.8
ZZ	2.1 ± 0.3	2.4 ± 0.4	3.2 ± 0.5	4.7 ± 0.7	12.3 ± 1.0
$\mathrm{Z} \gamma$	3.4 ± 1.3	0.4 ± 0.4	5.2 ± 1.8	0	9.1 ± 2.2
$\mathrm{W}\gamma ^*$	0	0	0	2.8 ± 1.0	2.8 ± 1.0
*VVV*	6.7 ± 2.2	8.7 ± 2.8	11.6 ± 3.8	14.8 ± 5.1	41.9 ± 7.3
Total background ($N_{\text{bkg}}$)	30.6 ± 13.0	43.5 ± 21.2	74.4 ± 33.3	84.7 ± 38.1	233.2 ± 56.3
WZ	211.1 ± 1.6	262.1 ± 1.8	346.7 ± 2.1	447.8 ± 2.4	1267.7 ± 4.0
Total expected	241.6 ± 13.1	305.7 ± 21.3	421.0 ± 33.3	532.4 ± 38.2	1500.8 ± 56.5
Data ($N_{\text{obs}}$)	258	298	435	568	1559

Independent samples are used to measure the efficiencies $\epsilon _{\mathrm{p}}$ and $\epsilon _{\mathrm{n}}$ [38]. The prompt lepton efficiency $\epsilon _{\mathrm{p}}$ is obtained from a $\mathrm{Z} \rightarrow \ell \ell$ sample, while the nonprompt lepton efficiency $\epsilon _{\mathrm{n}}$ is measured using a quantum chromodynamics (QCD) multijet sample. Events in this sample are triggered by a single lepton. The lepton selection used in these triggers is looser than the loose lepton selection referred to earlier in this section. The leading jet in the event is required to be well separated from the triggering lepton and have a transverse momentum larger than 50$\,\text{GeV}$ for the 7$\,\text{TeV}$ data sample, and larger than 35 (20)$\,\text{GeV}$ for the 8$\,\text{TeV}$ sample if the triggering lepton is an electron (muon). Events with leptons from $\mathrm{Z}$ decays are rejected by requiring exactly one lepton in the final state. To reject events with leptons from $\mathrm{W}$ decays, both the missing transverse energy and the $\mathrm{W}$ transverse mass are required to be less than 20$\,\text{GeV}$. This selection provides a clean sample to estimate the nonprompt lepton efficiency. Both efficiencies $\epsilon _{\mathrm{p}}$ and $\epsilon _{\mathrm{n}}$ are measured in several lepton $(p_{\mathrm{T}}, \eta )$ bins. For 7$\,\text{TeV}$  (8$\,\text{TeV}$) data, the measured nonprompt efficiencies for leptons are in the range 1–6% (1–10%), while they are in the range 1–5% (7–20%) for muons. The measured prompt efficiencies lie between 60 and 95% for electrons, and between 71 and 99% for muons for both the 7 and 8$\,\text{TeV}$ data samples.

The number of events with nonprompt leptons in each final state obtained with this method is given in Table 1. While these results include the contribution of events with any number of misidentified leptons, simulation studies show that the contribution from backgrounds with two or three misidentified leptons, such as W+jets or QCD multijet processes, is negligible, so the nonprompt lepton background is completely dominated by $\mathrm{t}\overline{\mathrm{t}}$ and Z+jets processes.

The remaining background is composed of events with three prompt leptons, such as the $ZZ\rightarrow 2\ell 2\ell '$ process in which one of the four final-state leptons has not been identified, as well as processes with three or more heavy bosons in the final states (*VVV*), and the $\mathrm{W}\gamma ^*$ process, with $\gamma ^*\rightarrow \ell ^+\ell ^-$. These backgrounds are estimated from simulation. The relevant W$\gamma ^*$ process is defined for low $\gamma ^*$ masses, $m_{\gamma ^*} < 12\,\text{GeV}$, so it does not overlap with the W$\gamma ^*$ process included in the signal simulation and it is simulated separately. It is considered a background since it does not fall in the fiducial phase space of the proposed measurement. Such W$\gamma ^*$ processes would be accepted by the event selection only if the charged lepton from the W decay is wrongly interpreted as coming from the $\mathrm{Z}/\gamma ^*$ decay. The contribution of $\mathrm{Z} \gamma$ events in which the photon is misidentified as a lepton is also determined from simulation. Prompt photons will not contribute to a nonprompt lepton signal since photons and electrons have a similar signature in the detector. Prompt photons in $\mathrm{Z} \gamma$ events will also typically be isolated from other final state particles.

**Fig. 2:**
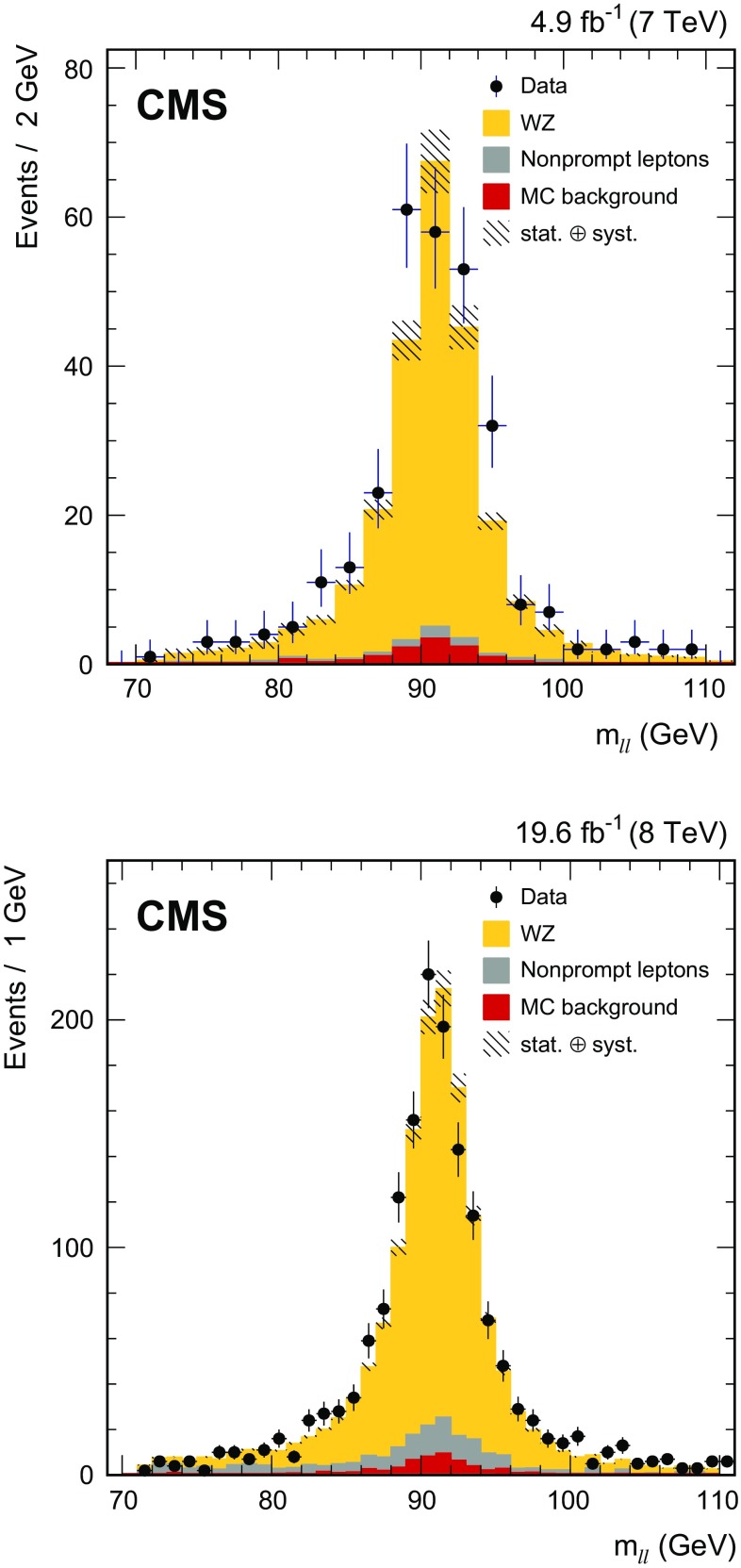
Distributions of the dilepton invariant mass $m_{\ell \ell }$ in the WZ candidate events in 7$\,\text{TeV}$ (*top*) and 8$\,\text{TeV}$ (*bottom*) data. *Points* represent data and the *shaded* histograms represent the WZ signal and the background processes. The contribution from nonprompt leptons, dominated by the $\mathrm{t}\overline{\mathrm{t}}$ and Z+jets production, is obtained from data control samples. The contribution from all other backgrounds, labeled ‘MC background’, as well as the signal contribution are determined from simulation

We finally consider the contribution of WZ decays, in which either the $\mathrm{W}$ or $\mathrm{Z}$ boson decays to a $\tau$ lepton. Such decays are considered a background to the signal. Their contribution is subtracted using the fraction of selected WZ decays that have $\tau$ leptons in the final state. This fraction, labeled $f_\tau$, is estimated from simulation for each of the four final states, and lies in between 6.5 and 7.6%. This background is almost entirely composed of WZ events with $\mathrm{W}\rightarrow \tau \nu$ decays where the $\tau$ lepton subsequently decays into an electron or a muon.

After applying all selection criteria, 293 (1559) events are selected from the 7 (8)$\,\text{TeV}$ data corresponding to an integrated luminosity of 4.9 (19.6)$\,\text{fb}^{-1}$. The yields for each leptonic channel, together with the expectations from MC simulation and data control samples are given in Table 1. The inclusive distributions of the dilepton invariant mass $m_{\ell \ell }$ for both 7 and 8$\,\text{TeV}$ data samples are shown in Fig. 2.

## Systematic uncertainties

Systematic uncertainties can be grouped into three categories: the determination of signal efficiency, the estimation of background yields, and the luminosity measurement.

The first group includes uncertainties affecting the signal efficiency, referred to as $\epsilon _\mathrm{{sig}}$, which accounts for both detector geometrical acceptance and reconstruction and selection efficiencies. It is determined from simulation. Uncertainties on $\epsilon _\mathrm{{sig}}$ depend on theoretical uncertainties in the PDFs. The PDF uncertainty is evaluated following the prescription in Ref. [39] using the CTEQ66 [26] PDF set. The uncertainties from normalization ($\mu _R$) and factorization ($\mu _F$) scales are estimated by varying both scales independently in the range ($0.5 \mu _0, 2\mu _0$) around their nominal value $\mu _0=0.5 (M_{\mathrm{Z}}+M_{\mathrm{W}})$ with the constraint $0.5\leq \mu _R / \mu _F \leq2$. The signal efficiency $\epsilon _{\text{sig}}$ is also affected by experimental uncertainties in the muon momentum scale and in the electron energy scale, lepton reconstruction and identification efficiencies, $E_{\mathrm{T}}^{\text{miss}}$ calibration scale, and pileup contributions. The effect of the muon momentum scale is estimated by varying the momentum of each muon in the simulated signal sample within the momentum scale uncertainty, which is 0.2% [32]. The same is done for electrons by varying the energy of reconstructed electrons within the uncertainty of the energy scale measurement, which is $p_{\mathrm{T}}$ and $\eta$ dependent and is typically below 1%. The signal efficiency $\epsilon _{\text{sig}}$ also depends on the uncertainties in the ratios of observed-to-simulated efficiencies of the lepton trigger, reconstruction, and identification requirements. These ratios are used in the determination of $\epsilon _{\text{sig}}$ to account for efficiency differences between data and simulation. They are varied within their uncertainties, which depend on the lepton $p_{\mathrm{T}}$ and $\eta$ and are about 1%. The uncertainty from the $E_{\mathrm{T}}^{\text{miss}}$ calibration is determined by scaling up and down the energy of all objects used for the $E_{\mathrm{T}}^{\text{miss}}$ determination within their uncertainties. Finally, $\epsilon _{\text{sig}}$ is affected by the uncertainty in the pileup contribution. Simulated events are reweighted to match the distribution of pileup interactions, which is estimated using a procedure that extracts the pileup from the instantaneous bunch luminosity and the total inelastic pp cross section. The weights applied to simulated events are changed by varying this cross section by 5% uncertainty [40].

**Table 2:** Summary of relative uncertainties, in units of percent, in the WZ cross section measurement at 7 and 8$\,\text{TeV}$

Source	$\sqrt{s} = 7\,\text{TeV}$				$\sqrt{s} = 8\,\text{TeV}$			
	$\mathrm{e}\mathrm{e}\mathrm{e}$	$\mathrm{e}\mathrm{e}\mu$	$\mu \mu \mathrm{e}$	$\mu \mu \mu$	$\mathrm{e}\mathrm{e}\mathrm{e}$	$\mathrm{e}\mathrm{e}\mu$	$\mu \mu \mathrm{e}$	$\mu \mu \mu$
Renorm. and fact. scales	1.3	1.3	1.3	1.3	3.0	3.0	3.0	3.0
PDFs	1.4	1.4	1.4	1.4	1.4	1.4	1.4	1.4
Pileup	0.3	0.5	1.0	0.6	0.2	0.4	0.3	0.2
Lepton and trigger efficiency	2.9	2.7	2.0	1.4	3.4	2.5	2.5	3.2
Muon momentum scale	–	0.6	0.4	1.1	–	0.5	0.8	1.3
Electron energy scale	1.9	0.8	1.2	–	1.4	0.8	0.8	–
$E_{\mathrm{T}}^{\text{miss}}$	3.7	3.4	4.3	3.7	1.5	1.5	1.6	1.2
ZZ cross section	0.5	0.9	0.6	0.9	0.1	0.1	0.1	0.1
$\mathrm{Z} \gamma$ cross section	0.0	0.0	0.1	0.0	0.2	0.0	0.2	0.0
$\mathrm{t}\overline{\mathrm{t}}$ and Z+jets	2.7	6.5	6.3	6.0	4.6	7.2	6.1	7.7
Other simulated backgrounds	0.2	0.2	0.9	0.2	1.0	1.1	1.1	1.0
Total systematic uncertainty	6.1	7.8	8.1	7.2	7.0	8.6	7.7	9.2
Statistical uncertainty	13.5	13.9	13.1	11.0	7.7	7.2	6.4	5.2
Integrated luminosity uncertainty	2.2	2.2	2.2	2.2	2.6	2.6	2.6	2.6

The second group comprises uncertainties in the background yield. The uncertainty in the background from nonprompt leptons [38] is estimated by varying the leading jet $p_{\mathrm{T}}$ threshold used to select the control sample of misidentified leptons, since the energy of the leading jet determines the composition of the sample. The uncertainties from other background processes, whose contributions are determined from simulation, are calculated by varying their predicted cross sections within uncertainties. The cross sections are varied by 15% (14%) for $\mathrm{ZZ}$, by 15% (7%) for $\mathrm{Z} \gamma$, by 50% (50%) for the $\mathrm{V} \mathrm{V} \mathrm{V}$ processes, and by 20% for $\mathrm{W}\gamma ^*$ for the 8$\,\text{TeV}$  (7$\,\text{TeV}$) measurements, based on the uncertainties of the measurements of these processes [41–45].

Finally, the uncertainty in the measurement of the integrated luminosity is 2.2 (2.6)% for 7 (8)$\,\text{TeV}$ data [46, 47].

A summary of all uncertainties is given in Table 2.

## Results

### Inclusive cross section measurement

The inclusive WZ cross section $\sigma ( \mathrm{p}\mathrm{p}\rightarrow \mathrm{W}\mathrm{Z} +X)$ in the $\ell \nu \ell '\ell '$ final state is related to the number of observed events in that final state, $N_{\text{obs}}$, through the following expression,

$$\begin{aligned}&\sigma (\mathrm{p}\mathrm{p}\rightarrow {\mathrm{W}\mathrm{Z} +X}) \, \mathcal {B} ({\mathrm{W}}\rightarrow \ell \nu ) \, \mathcal {B} ({\mathrm{Z}}\rightarrow \ell '\ell ')\\&\quad = \left( 1 - f_{\tau }\right) \frac{N_\text{obs} - N_\text{bkg}}{\epsilon _{\text{sig}}\,\mathcal {L}}, \end{aligned}$$

where $\mathcal {B} ({\mathrm{W}}\rightarrow \ell \nu )$ and $\mathcal {B} ({\mathrm{Z}}\rightarrow \ell '\ell ')$ are the $\mathrm{W}$ and $\mathrm{Z}$ boson leptonic branching fractions per lepton species, and $f_{\tau }$ accounts for the expected fraction of selected $\mathrm{W}\mathrm{Z} \rightarrow \ell \nu \ell '\ell '$ decays produced through at least one prompt $\tau$ decay in the final state after removing all other backgrounds. The number of expected background events is $N_\text{bkg}$, and the number of signal events is determined by subtracting $N_\text{bkg}$ from the observed data $N_\text{obs}$. The signal efficiency $\epsilon _{\text{sig}}$ accounts for both detector geometrical acceptance and reconstruction and selection efficiencies. It is obtained for each of the four final states using the simulated WZ sample by calculating the ratio of the number of events passing the full selection to the number of generated $\mathrm{W}\mathrm{Z} \rightarrow \ell \nu \ell '\ell '$ events with $71< m_{\ell '\ell '} < 111\,\text{GeV}$, where $m_{\ell '\ell '}$ is the dilepton mass of the two leptons from the Z boson decay prior to final state photon radiation. Only events decaying into the respective final state are considered in both the numerator and denominator of this fraction. The resulting cross section values are reported in Table 3 for the four leptonic channels. There is good agreement among the four channels for both the 7 and 8$\,\text{TeV}$ data.

**Table 3:** Measured WZ cross section in the four leptonic channels at $\sqrt{s} = 7$ and 8$\,\text{TeV}$

Channel	$\sigma ({\mathrm{p}\mathrm{p}}\rightarrow {\mathrm{W}\mathrm{Z}};\ \sqrt{s} = 7\,\text{TeV})$ [pb]
$\mathrm{e}\mathrm{e}\mathrm{e}$	$22.46 \pm 3.12\,\text{(stat)} \pm 0.43\,\text{(theo)} \pm 1.33\,\text{(exp)} \pm 0.49\,\text{(lumi)}$
$\mathrm{e}\mathrm{e}\mu$	$19.04 \pm 2.75\,\text{(stat)} \pm 0.36 ( {\mathrm{theo}}) \pm 1.50\,\text{(exp)} \pm 0.42\,\text{(lumi)}$
$\mu \mu \mathrm{e}$	$19.13 \pm 2.60\,\text{(stat)} \pm 0.37\,\text{(theo)} \pm 1.56\,\text{(exp)} \pm 0.42\,\text{(lumi)}$
$\mu \mu \mu$	$20.36 \pm 2.31\,\text{(stat)} \pm 0.39\,\text{(theo)} \pm 1.48\,\text{(exp)} \pm 0.45\,\text{(lumi)}$

Channel	$\sigma ({\mathrm{p}\mathrm{p}}\rightarrow {\mathrm{W}\mathrm{Z}};\ \sqrt{s} = 8\,\text{TeV})$ [pb]
eee	$24.80 \pm 1.92\,\text{(stat)} \pm 0.82 ( {\mathrm{theo}}) \pm 1.53\,\text{(exp)} \pm 0.64\,\text{(lumi)}$
$\mathrm{e}\mathrm{e}\mu$	$22.38 \pm 1.62\,\text{(stat)} \pm 0.74 ( {\mathrm{theo}}) \pm 1.78 \,\text{(exp)} \pm 0.58\,\text{(lumi)}$
$\mu \mu \mathrm{e}$	$23.94 \pm 1.52\,\text{(stat)} \pm 0.79 ( {\mathrm{theo}}) \pm 1.66\,\text{(exp)} \pm 0.62\,\text{(lumi)}$
$\mu \mu \mu$	$24.93 \pm 1.29\,\text{(stat)} \pm 0.83 ( {\mathrm{theo}}) \pm 2.14 \,\text{(exp)} \pm 0.65\,\text{(lumi)}$

These four measurements are combined using the best linear unbiased estimator (BLUE) method [48]. We have assumed full correlation for all uncertainties common to different channels. Combining the four leptonic channels, the total WZ cross section for $71< m_{\mathrm{Z}} < 111\,\text{GeV}$, at 7 and 8$\,\text{TeV}$, is measured to be

$$\begin{aligned}&\sigma ({\mathrm{p}\mathrm{p}}\rightarrow {\mathrm{W}\mathrm{Z}};~\sqrt{s} = 7\,\text{TeV})\\&\quad = 20.14 \pm 1.32\,\text{(stat)} \pm 0.38\,\text{(theo)} \pm 1.06\,\text{(exp)} \\&\quad \pm 0.44\,\text{(lumi) pb}. \\&\sigma ({\mathrm{p}\mathrm{p}}\rightarrow {\mathrm{W}\mathrm{Z}};~\sqrt{s} = 8\,\text{TeV})\\&\quad = 24.09 \pm 0.87\,\text{(stat)} \pm 0.80\,\text{(theo)} \pm 1.40\,\text{(exp)} \\&\quad \pm 0.63\,\text{(lumi) pb}. \end{aligned}$$

**Fig. 3:**
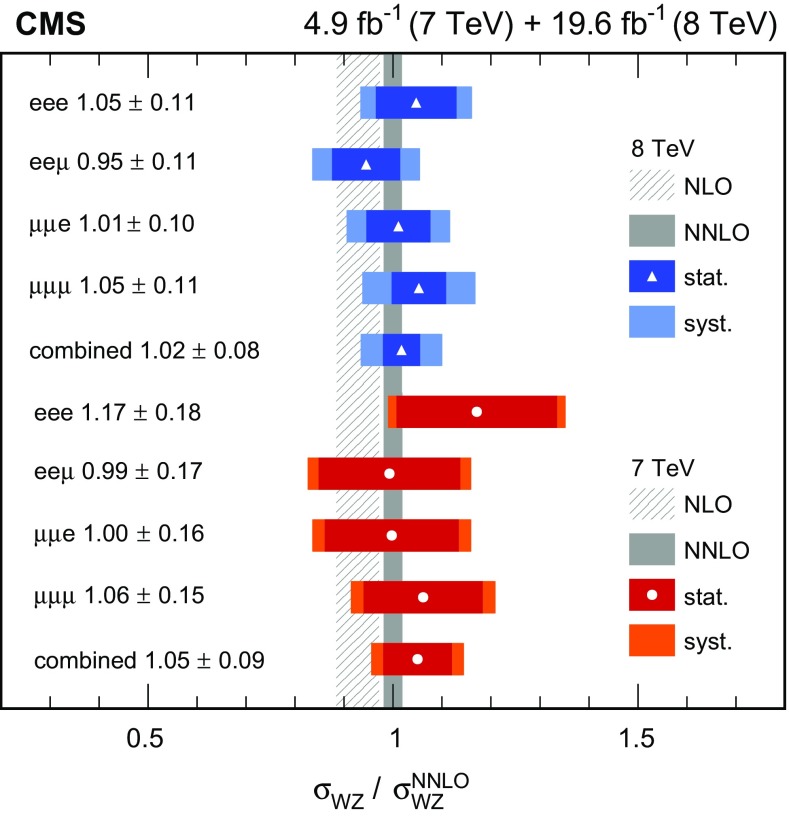
Ratio of measured inclusive cross sections to NNLO predictions. The *vertical gray* bands represent the theoretical uncertainties at 7 and 8$\,\text{TeV}$

These results can be compared with recent calculations at NLO and next-to-next-to-leading order (NNLO) in QCD via Matrix [49]. The NLO (NNLO) predictions are $17.72^{+5.3\%}_{-1.8\%}$ ($19.18_{-1.8\%}^{+1.7\%}$)$\,\text{pb}$ at 7$\,\text{TeV}$, and $21.80^{+5.1\%}_{-3.9\%}$ ($23.68 \pm 1.8\%$)$\,\text{pb}$ at 8$\,\text{TeV}$, where uncertainties include only scale variations. All these predictions are in agreement with the measured values within uncertainties. The NLO predictions are slightly lower than the measured values, and a better agreement is observed for the NNLO observations at both centre-of-mass energies. The ratios of the inclusive cross sections for the individual and combined results to the NLO and NNLO predictions are shown in Fig. 3.

**Fig. 4:**
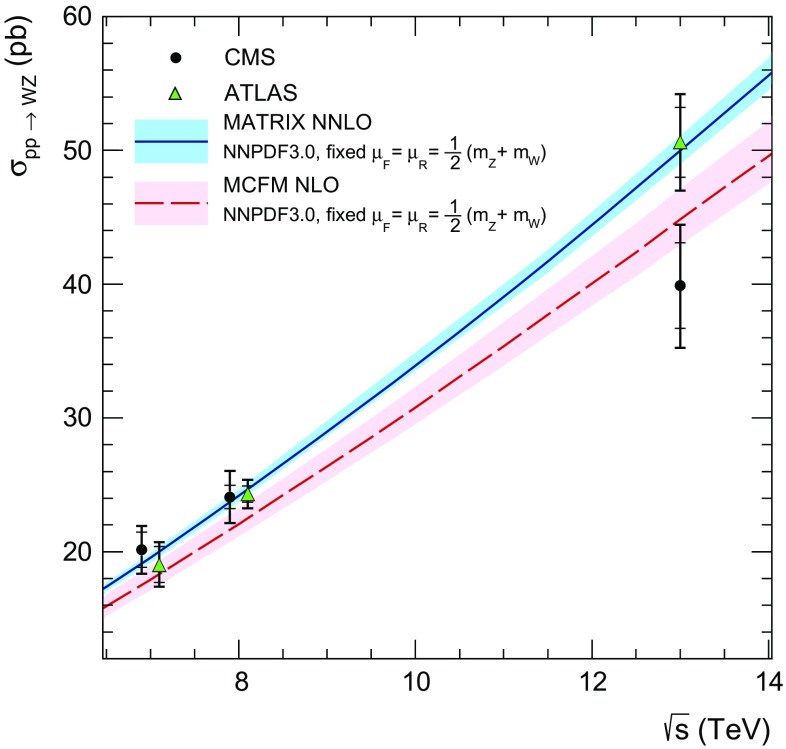
The WZ total cross section as a function of the proton–proton centre-of-mass energy. Results from the CMS and ATLAS experiments are compared to the predictions of MCFM and Matrix. The data uncertainties are statistical (*inner bars*) and statistical plus systematic added in quadrature (*outer bars*). The uncertainties covered by the band around the theoretical predictions are described in the text. The theoretical predictions and the CMS 13$\,\text{TeV}$ cross section are calculated for the Z boson mass window 60–120$\,\text{GeV}$. The CMS 7 and 8$\,\text{TeV}$ cross sections presented in this paper are calculated for the Z boson mass window 71–111$\,\text{GeV}$ (estimated correction factor 2%), while all ATLAS measurements are performed with the Z boson mass window 66–116$\,\text{GeV}$ (1%)

The total WZ production cross sections for different centre-of-mass energies from the CMS [13] and ATLAS [10–12] experiments are compared to theoretical predictions calculated with MCFM (NLO) and Matrix (NNLO) in Fig. 4. The theoretical predictions describe, within the uncertainties, the energy dependence of the measured cross sections. The band around the theoretical predictions in this figure reflects uncertainties generated by varying the factorization and renormalization scales up and down by a factor of two and also the (PDF+$\alpha _\mathrm {S}$) uncertainty of NNPDF3.0 for NLO predictions.

**Table 4:** Differential WZ cross section as a function of the Z transverse momentum at $\sqrt{s}=8\,\text{TeV}$ for the four leptonic final states. The first uncertainty is statistical, the second is systematic, and the third is the integrated luminosity

$p_{\mathrm{T}} ^{\mathrm{Z}}$ [$\text{GeV}$ ]	$\mathrm{d}\sigma / \mathrm{d}p_{\mathrm{T}} ^{\mathrm{Z}}$ [pb /$\text{GeV}$ ]			
	$\mathrm{e}\mathrm{e}\mathrm{e}$	$\mathrm{e}\mathrm{e}\mu$	$\mu \mu \mathrm{e}$	$\mu \mu \mu$
0–20	$$\begin{array}{lll} (1.63 &{} \pm &{} 0.90 \\ &{} \pm &{} 0.22 \\ &{} \pm &{} 0.04)\\ &{} &{} \times 10 ^{-1} \end{array}$$	$$\begin{array}{lll} (9.3 &{} \pm &{}6.8 \\ &{} \pm &{} 1.3 \\ &{} \pm &{} 0.2)\\ &{} &{} \times 10 ^{-2} \end{array}$$	$$\begin{array}{lll} (1.68 &{} \pm &{} 0.92 \\ &{} \pm &{} 0.21 \\ &{} \pm &{} 0.04)\\ &{} &{} \times 10 ^{-1} \end{array}$$	$$\begin{array}{lll} (2.01 &{} \pm &{} 1.00 \\ &{} \pm &{} 0.20 \\ &{} \pm &{} 0.05)\\ &{} &{} \times 10 ^{-1} \end{array}$$
20–40	$$ \begin{array}{lll} (3.9 &{} \pm &{} 1.4 \\ &{} \pm &{} 0.5 \\ &{} \pm &{} 0.1)\\ &{} &{} \times 10 ^{-1} \end{array} $$	$$ \begin{array}{lll} (3.17 &{} \pm &{}1.26 \\ &{} \pm &{} 0.39 \\ &{} \pm &{} 0.08)\\ &{} &{} \times 10 ^{-1} \end{array} $$	$$ \begin{array}{lll} (2.76 &{} \pm &{} 1.18 \\ &{} \pm &{} 0.62 \\ &{} \pm &{} 0.07)\\ &{} &{} \times 10 ^{-1} \end{array} $$	$$ \begin{array}{lll} (3.42 &{} \pm &{} 1.31 \\ &{} \pm &{} 0.57 \\ &{} \pm &{} 0.09)\\ &{} &{} \times 10 ^{-1} \end{array} $$
40–60	$$ \begin{array}{lll} (3.14 &{} \pm &{} 1.25 \\ &{} \pm &{} 0.60 \\ &{} \pm &{} 0.08)\\ &{} &{} \times 10 ^{-1} \end{array} $$	$$ \begin{array}{lll} (2.70 &{} \pm &{}1.16 \\ &{} \pm &{} 0.43 \\ &{} \pm &{} 0.07)\\ &{} &{} \times 10 ^{-1} \end{array} $$	$$ \begin{array}{lll} (2.29 &{} \pm &{} 1.07 \\ &{} \pm &{} 0.48 \\ &{} \pm &{} 0.06)\\ &{} &{} \times 10 ^{-1} \end{array} $$	$$ \begin{array}{lll} (2.82 &{} \pm &{} 1.19 \\ &{} \pm &{} 0.56 \\ &{} \pm &{} 0.07)\\ &{} &{} \times 10 ^{-1} \end{array} $$
60–80	$$ \begin{array}{lll} (1.69 &{} \pm &{} 0.92 \\ &{} \pm &{} 0.30 \\ &{} \pm &{} 0.04)\\ &{} &{} \times 10 ^{-1} \end{array} $$	$$ \begin{array}{lll} (2.07 &{} \pm &{}1.02 \\ &{} \pm &{} 0.31 \\ &{} \pm &{} 0.05)\\ &{} &{} \times 10 ^{-1} \end{array} $$	$$ \begin{array}{lll} (2.31 &{} \pm &{} 1.07 \\ &{} \pm &{} 0.33 \\ &{} \pm &{} 0.06)\\ &{} &{} \times 10 ^{-1} \end{array} $$	$$ \begin{array}{lll} (2.03 &{} \pm &{} 1.01 \\ &{} \pm &{} 0.31 \\ &{} \pm &{} 0.05)\\ &{} &{} \times 10 ^{-1} \end{array} $$
80–100	$$ \begin{array}{lll} (1.27 &{} \pm &{} 0.80 \\ &{} \pm &{} 0.23 \\ &{} \pm &{} 0.03)\\ &{} &{} \times 10 ^{-1} \end{array} $$	$$ \begin{array}{lll} (1.02 &{} \pm &{}0.71 \\ &{} \pm &{} 0.17 \\ &{} \pm &{} 0.03)\\ &{} &{} \times 10 ^{-1} \end{array} $$	$$ \begin{array}{lll} (1.30 &{} \pm &{} 0.81 \\ &{} \pm &{} 0.25 \\ &{} \pm &{} 0.03)\\ &{} &{} \times 10 ^{-1} \end{array} $$	$$ \begin{array}{lll} (1.25 &{} \pm &{} 0.79 \\ &{} \pm &{} 0.21 \\ &{} \pm &{} 0.03)\\ &{} &{} \times 10 ^{-1} \end{array} $$
100–120	$$ \begin{array}{lll} (8.1 &{} \pm &{} 6.4 \\ &{} \pm &{} 2.2 \\ &{} \pm &{} 0.2)\\ &{} &{} \times 10 ^{-2} \end{array} $$	$$ \begin{array}{lll} (2.76 &{} \pm &{}3.72 \\ &{} \pm &{} 1.55 \\ &{} \pm &{} 0.07)\\ &{} &{} \times 10 ^{-2} \end{array} $$	$$ \begin{array}{lll} (5.0 &{} \pm &{} 5.0 \\ &{} \pm &{} 1.4 \\ &{} \pm &{} 0.1)\\ &{} &{} \times 10 ^{-2} \end{array} $$	$$ \begin{array}{lll} (7.8 &{} \pm &{} 6.3 \\ &{} \pm &{} 1.4 \\ &{} \pm &{} 0.2)\\ &{} &{} \times 10 ^{-2} \end{array} $$
120–140	$$ \begin{array}{lll} (5.8 &{} \pm &{} 5.4 \\ &{} \pm &{} 0.9 \\ &{} \pm &{} 0.1)\\ &{} &{} \times 10 ^{-2} \end{array} $$	$$ \begin{array}{lll} (6.2 &{} \pm &{}5.6 \\ &{} \pm &{} 0.8 \\ &{} \pm &{} 0.2)\\ &{} &{} \times 10 ^{-2} \end{array} $$	$$ \begin{array}{lll} (3.12 &{} \pm &{} 3.95 \\ &{} \pm &{} 1.13 \\ &{} \pm &{} 0.08)\\ &{} &{} \times 10 ^{-2} \end{array} $$	$$ \begin{array}{lll} (4.1 &{} \pm &{} 4.5 \\ &{} \pm &{} 1.2 \\ &{} \pm &{} 0.1)\\ &{} &{} \times 10 ^{-2} \end{array} $$
140–200	$$ \begin{array}{lll} (1.07 &{} \pm &{} 1.34 \\ &{} \pm &{} 0.58 \\ &{} \pm &{} 0.03)\\ &{} &{} \times 10 ^{-2} \end{array} $$	$$ \begin{array}{lll} (1.09 &{} \pm &{}1.35 \\ &{} \pm &{} 0.62 \\ &{} \pm &{} 0.03)\\ &{} &{} \times 10 ^{-2} \end{array} $$	$$ \begin{array}{lll} (2.73 &{} \pm &{} 2.13 \\ &{} \pm &{} 0.56 \\ &{} \pm &{} 0.07)\\ &{} &{} \times 10 ^{-2} \end{array} $$	$$ \begin{array}{lll} (1.46 &{} \pm &{} 1.56 \\ &{} \pm &{} 0.53 \\ &{} \pm &{} 0.04)\\ &{} &{} \times 10 ^{-2} \end{array} $$
200–300	$$ \begin{array}{lll} (3.66 &{} \pm &{} 6.05 \\ &{} \pm &{} 1.58 \\ &{} \pm &{} 0.10)\\ &{} &{} \times 10 ^{-3} \end{array} $$	$$ \begin{array}{lll} (9.0 &{} \pm &{}9.5 \\ &{} \pm &{} 1.7 \\ &{} \pm &{} 0.2)\\ &{} &{} \times 10 ^{-3} \end{array} $$	$$ \begin{array}{lll} (7.4 &{} \pm &{} 8.6 \\ &{} \pm &{} 1.7 \\ &{} \pm &{} 0.2)\\ &{} &{} \times 10 ^{-3} \end{array} $$	$$ \begin{array}{lll} (5.8 &{} \pm &{} 7.6 \\ &{} \pm &{} 1.8 \\ &{} \pm &{} 0.2)\\ &{} &{} \times 10 ^{-3} \end{array} $$

### Differential cross section measurement

Using the larger available integrated luminosity in the 8$\,\text{TeV}$ sample, we measure the differential WZ cross sections as a function of three different observables: the Z boson $p_{\mathrm{T}}$, the number of jets produced in association with the $\ell \nu \ell '\ell '$ final state, and the $p_{\mathrm{T}}$ of the leading accompanying jet. For the latter two measurements, the differential cross sections are defined for generated jets built from all stable particles using the anti-$k_{\mathrm{T}}$ algorithm [35] with a distance parameter of 0.5, but excluding the electrons, muons, and neutrinos from the $\mathrm{W}$ and $\mathrm{Z}$ boson decays. Jets are required to have $p_{\mathrm{T}} > 30\,\text{GeV}$ and $|\eta |<2.5$. They also must be separated from the charged leptons from the W and Z boson decays by $\Delta R(\text{jet},\ell )>0.5$. The jets reconstructed from PF candidates, clustered by the same algorithm, have to fulfill the same requirements.

To obtain the cross section in each bin, the background contribution is first subtracted from the observed yield in each bin, in the same way as it was done for the inclusive cross section. The measured signal spectra are then corrected for the detector effects. These include efficiencies as well as bin-to-bin migrations due to finite resolution. Both effects are treated using the iterative D’Agostini unfolding technique [50], as implemented in RooUnfold [51], with 5 iterations. The technique uses response matrices that relate the true distribution of an observable to the observed distribution after including detector effects. The response matrices are obtained using the signal MC sample for all four leptonic final states separately. The unfolded spectra are then used to obtain differential cross sections for all four leptonic final states. The four channels are combined bin-by-bin.

**Fig. 5:**
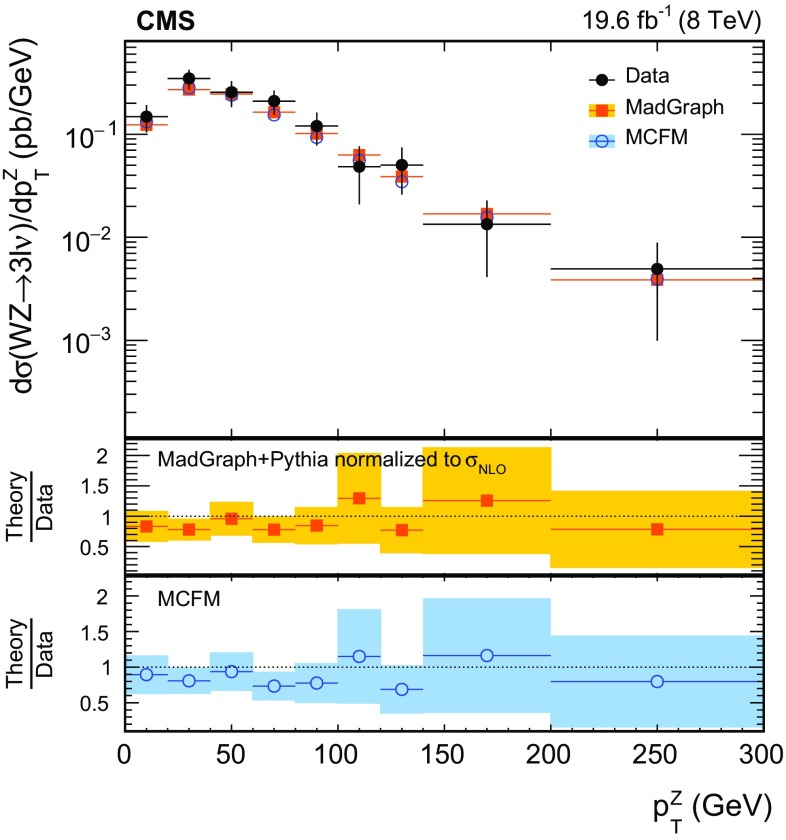
Differential WZ cross section at $\sqrt{s}=8\,\text{TeV}$ as a function of the Z boson transverse momentum. The measurement is compared with mcfm and MadGraph predictions. The MadGraph prediction is rescaled to the total NLO cross section as predicted by mcfm. The error bands in the ratio plots indicate the relative errors on the data in each bin and contain both statistical and systematic uncertainties

**Fig. 6:**
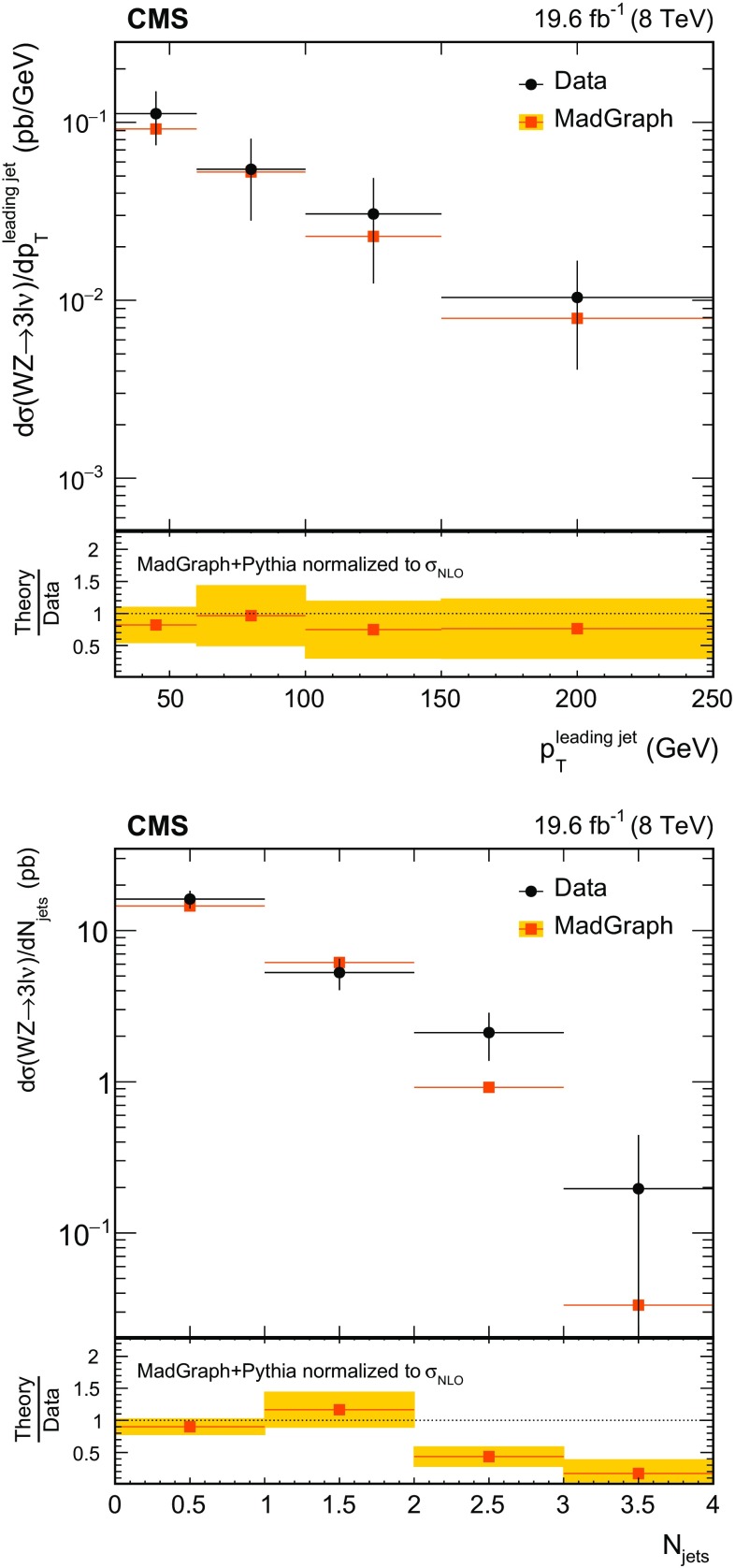
Differential WZ cross section at $\sqrt{s}=8\,\text{TeV}$ as a function of: (*top*) the leading jet transverse momentum; (*bottom*) the number of accompanying jets. The measurements are compared with MadGraph predictions. The MadGraph prediction is rescaled to the total NLO cross section as predicted by mcfm. The error bands in the ratio plots indicate the relative errors on the data in each bin and contain both statistical and systematic uncertainties

A few additional sources of systematic uncertainties need to be considered with respect to those described in Sect. 6. The measurements involving jets are affected by the experimental uncertainties in the jet energy scale and resolution. The effects on the response matrices are studied by smearing and scaling the jet energies within their uncertainties. Furthermore, an uncertainty due to the limited size of the simulated sample used to build the response matrices is also included. The unfolding procedure introduces statistical correlations between bins, which range from a few percent up to 40% in a few cases. These correlations are taken into account together with correlated systematic uncertainties by using a generalization of the BLUE method as described in Ref. [52]. The three measured differential cross sections are given in Tables 4, 5, and 6 for each of the four final states, and the combined results are given in Table 7. The combined differential cross sections are shown in Figs. 5 and 6.

**Table 5:** Differential WZ cross section as a function of the jet multiplicity at $\sqrt{s}=8\,\text{TeV}$ for the four leptonic final states. Notations are as in Table 4

$N_\text{jets}$	$\mathrm{d}\sigma / \mathrm{d}N_\text{jets}$ [pb]			
	$\mathrm{e}\mathrm{e}\mathrm{e}$	$\mathrm{e}\mathrm{e}\mu$	$\mu \mu \mathrm{e}$	$\mu \mu \mu$
0 Jets	$$ \begin{array}{lll} 16.60 &{} \pm &{} 4.07 \\ &{} \pm &{} 1.04 \\ &{} \pm &{} 0.43 \end{array} $$	$$ \begin{array}{lll} 15.68 &{} \pm &{} 3.96 \\ &{} \pm &{} 1.03 \\ &{} \pm &{} 0.41 \end{array} $$	$$ \begin{array}{lll} 14.97 &{} \pm &{} 3.87 \\ &{} \pm &{} 0.93 \\ &{} \pm &{} 0.39 \end{array} $$	$$ \begin{array}{lll} 18.78 &{} \pm &{} 4.33 \\ &{} \pm &{} 1.11\\ &{} \pm &{} 0.49 \end{array} $$
1 Jet	$$ \begin{array}{lll} 6.06 &{} \pm &{} 2.46 \\ &{} \pm &{} 0.48 \\ &{} \pm &{} 0.16 \end{array} $$	$$ \begin{array}{lll} 4.80 &{} \pm &{} 2.19 \\ &{} \pm &{} 0.57 \\ &{} \pm &{} 0.12 \end{array} $$	$$ \begin{array}{lll} 5.32 &{} \pm &{} 2.31 \\ &{} \pm &{} 0.61 \\ &{} \pm &{} 0.14 \end{array} $$	$$ \begin{array}{lll} 4.84 &{} \pm &{} 2.20 \\ &{} \pm &{} 0.72\\ &{} \pm &{} 0.13 \end{array} $$
2 Jets	$$ \begin{array}{lll} 2.43 &{} \pm &{} 1.56 \\ &{} \pm &{} 0.34 \\ &{} \pm &{} 0.06 \end{array} $$	$$ \begin{array}{lll} 1.75 &{} \pm &{} 1.32 \\ &{} \pm &{} 0.32 \\ &{} \pm &{} 0.05 \end{array} $$	$$ \begin{array}{lll} 2.93 &{} \pm &{} 1.71 \\ &{} \pm &{} 0.26 \\ &{} \pm &{} 0.08 \end{array} $$	$$ \begin{array}{lll} 1.54 &{} \pm &{} 1.24 \\ &{} \pm &{} 0.32\\ &{} \pm &{} 0.04 \end{array} $$
3 Jets	$$ \begin{array}{lll} (7.8 &{} \pm &{} 27.9 \\ &{} \pm &{} 7.3 \\ &{} \pm &{} 0.2)\\ &{} &{} \times 10 ^{-2} \end{array} $$	$$ \begin{array}{lll} 0.45 &{} \pm &{} 0.67 \\ &{} \pm &{} 0.17 \\ &{} \pm &{} 0.01 \end{array} $$	$$ \begin{array}{lll} 0.42 &{} \pm &{} 0.65 \\ &{} \pm &{} 0.21 \\ &{} \pm &{} 0.01 \end{array} $$	$$ \begin{array}{lll} 0.79 &{} \pm &{} 0.89 \\ &{} \pm &{} 0.26\\ &{} \pm &{} 0.02 \end{array} $$

**Table 6:** Differential WZ cross section as a function of the leading jet transverse momentum at $\sqrt{s}=8\,\text{TeV}$ for the four leptonic final states. Notations are as in Table 4

$p_{\mathrm{T}} ^{\text{leading jet}}$ [$\text{GeV}$ ]	$\mathrm{d}\sigma / \mathrm{d}p_{\mathrm{T}} ^\text{leading jet}$ [pb/$\text{GeV}$ ]			
	$\mathrm{e}\mathrm{e}\mathrm{e}$	$\mathrm{e}\mathrm{e}\mu$	$\mu \mu \mathrm{e}$	$\mu \mu \mu$
30–60	$$ \begin{array}{lll} (1.22 &{} \pm &{} 0.64 \\ &{} \pm &{} 0.34 \\ &{} \pm &{} 0.03)\\ &{} &{} \times 10 ^{-1} \end{array} $$	$$ \begin{array}{lll} (1.11 &{} \pm &{}0.61 \\ &{} \pm &{} 0.20 \\ &{} \pm &{} 0.03)\\ &{} &{} \times 10 ^{-1} \end{array} $$	$$ \begin{array}{lll} (1.10 &{} \pm &{} 0.61 \\ &{} \pm &{} 0.24 \\ &{} \pm &{} 0.03)\\ &{} &{} \times 10 ^{-1} \end{array} $$	$$ \begin{array}{lll} (1.02 &{} \pm &{} 0.58 \\ &{} \pm &{} 0.24 \\ &{} \pm &{} 0.03)\\ &{} &{} \times 10 ^{-1} \end{array} $$
60–100	$$ \begin{array}{lll} (5.4 &{} \pm &{} 3.7 \\ &{} \pm &{} 1.7 \\ &{} \pm &{} 0.1)\\ &{} &{} \times 10 ^{-2} \end{array} $$	$$ \begin{array}{lll} (4.3 &{} \pm &{}3.3 \\ &{} \pm &{} 2.1 \\ &{} \pm &{} 0.1)\\ &{} &{} \times 10 ^{-2} \end{array} $$	$$ \begin{array}{lll} (6.5 &{} \pm &{} 4.0 \\ &{} \pm &{} 2.0 \\ &{} \pm &{} 0.2)\\ &{} &{} \times 10 ^{-2} \end{array} $$	$$ \begin{array}{lll} (6.3 &{} \pm &{} 4.0 \\ &{} \pm &{} 2.3 \\ &{} \pm &{} 0.2)\\ &{} &{} \times 10 ^{-2} \end{array} $$
100–150	$$ \begin{array}{lll} (2.96 &{} \pm &{} 2.43 \\ &{} \pm &{} 1.57 \\ &{} \pm &{} 0.08)\\ &{} &{} \times 10 ^{-2} \end{array} $$	$$ \begin{array}{lll} (3.26 &{} \pm &{}2.55 \\ &{} \pm &{} 1.40 \\ &{} \pm &{} 0.08)\\ &{} &{} \times 10 ^{-2} \end{array} $$	$$ \begin{array}{lll} (3.9 &{} \pm &{} 2.8 \\ &{} \pm &{} 1.2 \\ &{} \pm &{} 0.1)\\ &{} &{} \times 10 ^{-2} \end{array} $$	$$ \begin{array}{lll} (2.44 &{} \pm &{} 2.21 \\ &{} \pm &{} 1.32 \\ &{} \pm &{} 0.06)\\ &{} &{} \times 10 ^{-2} \end{array} $$
150–250	$$ \begin{array}{lll} (1.18 &{} \pm &{} 1.09 \\ &{} \pm &{} 0.29 \\ &{} \pm &{} 0.03)\\ &{} &{} \times 10 ^{-2} \end{array} $$	$$ \begin{array}{lll} (8.1 &{} \pm &{}9.0 \\ &{} \pm &{} 3.4 \\ &{} \pm &{} 0.2)\\ &{} &{} \times 10 ^{-3} \end{array} $$	$$ \begin{array}{lll} (1.07 &{} \pm &{} 1.03 \\ &{} \pm &{} 0.61 \\ &{} \pm &{} 0.03)\\ &{} &{} \times 10 ^{-2} \end{array} $$	$$ \begin{array}{lll} (1.00 &{} \pm &{} 1.00 \\ &{} \pm &{} 0.42 \\ &{} \pm &{} 0.03)\\ &{} &{} \times 10 ^{-2} \end{array} $$

**Table 7:** Combined result for the differential WZ cross sections at $\sqrt{s}=8\,\text{TeV}$

$p_{\mathrm{T}} ^{\mathrm{Z}}$ [$\text{GeV}$ ]	$\mathrm{d}\sigma / \mathrm{d}p_{\mathrm{T}} ^{\mathrm{Z}}$ [pb/$\text{GeV}$ ]
0–20	[1.48 ± 0.40$\,\text{(stat)}$ ± 0.17$\,\text{(syst)}$ ± 0.04$\,\text{(lumi)}$ ]$\times 10 ^{-1}$
20–40	[3.47 ± 0.60$\,\text{(stat)}$ ± 0.50$\,\text{(syst)}$ ± 0.09$\,\text{(lumi)}$ ]$\times 10 ^{-1}$
40–60	[2.56 ± 0.54$\,\text{(stat)}$ ± 0.49$\,\text{(syst)}$ ± 0.07$\,\text{(lumi)}$ ]$\times 10 ^{-1}$
60–80	[2.10 ± 0.47$\,\text{(stat)}$ ± 0.30$\,\text{(syst)}$ ± 0.05$\,\text{(lumi)}$ ]$\times 10 ^{-1}$
80–100	[1.20 ± 0.37$\,\text{(stat)}$ ± 0.21$\,\text{(syst)}$ ± 0.03$\,\text{(lumi)}$ ]$\times 10 ^{-1}$
100–120	[4.9 ± 2.3 $\,\text{(stat)}$ ± 1.5 $\,\text{(syst)}$ ± 0.1 $\,\text{(lumi)}$ ]$\times 10 ^{-2}$
120–140	[5.0 ± 2.2 $\,\text{(stat)}$ ± 1.0 $\,\text{(syst)}$ ± 0.1 $\,\text{(lumi)}$ ]$\times 10 ^{-2}$
140–200	[1.34 ± 0.73$\,\text{(stat)}$ ± 0.57$\,\text{(syst)}$ ± 0.03$\,\text{(lumi)}$ ]$\times 10 ^{-2}$
200–300	[4.9 ± 3.6 $\,\text{(stat)}$ ± 1.6 $\,\text{(syst)}$ ± 0.1 $\,\text{(lumi)}$ ]$\times 10 ^{-3}$

$N_\text{jets}$	$\mathrm{d}\sigma / \mathrm{d}N_\text{jets}$ [pb]
0 Jets	16.15 ± 1.95 $\,\text{(stat)}$ ± 0.88 $\,\text{(syst)}$ ± 0.42 $\,\text{(lumi)}$
1 Jet	5.27 ± 1.11 $\,\text{(stat)}$ ± 0.52 $\,\text{(syst)}$ ± 0.14 $\,\text{(lumi)}$
2 Jets	2.11 ± 0.69 $\,\text{(stat)}$ ± 0.27 $\,\text{(syst)}$ ± 0.05 $\,\text{(lumi)}$
3 Jets	0.196 ± 0.227$\,\text{(stat)}$ ± 0.102$\,\text{(syst)}$ ± 0.005$\,\text{(lumi)}$

$p_{\mathrm{T}} ^\text{leading jet}$ [$\text{GeV}$ ]	$\mathrm{d}\sigma / \mathrm{d}p_{\mathrm{T}} ^\text{leading jet}$ [pb/$\text{GeV}$ ]
30–60	[1.12 ± 0.30$\,\text{(stat)}$ ± 0.23$\,\text{(syst)}$ ± 0.03$\,\text{(lumi)}$ ]$\times 10 ^{-1}$
60–100	[5.5 ± 1.8 $\,\text{(stat)}$ ± 1.9 $\,\text{(syst)}$ ± 0.1 $\,\text{(lumi)}$ ]$\times 10 ^{-2}$
100–150	[3.06 ± 1.20$\,\text{(stat)}$ ± 1.37$\,\text{(syst)}$ ± 0.08$\,\text{(lumi)}$ ]$\times 10 ^{-2}$
150–250	[1.04 ± 0.48$\,\text{(stat)}$ ± 0.41$\,\text{(syst)}$ ± 0.03$\,\text{(lumi)}$ ]$\times 10 ^{-2}$

The differential cross sections are compared with the mcfm and MadGraph predictions. The MadGraph spectra are normalized to the NLO cross section as predicted by MCFM.

**Fig. 7:**
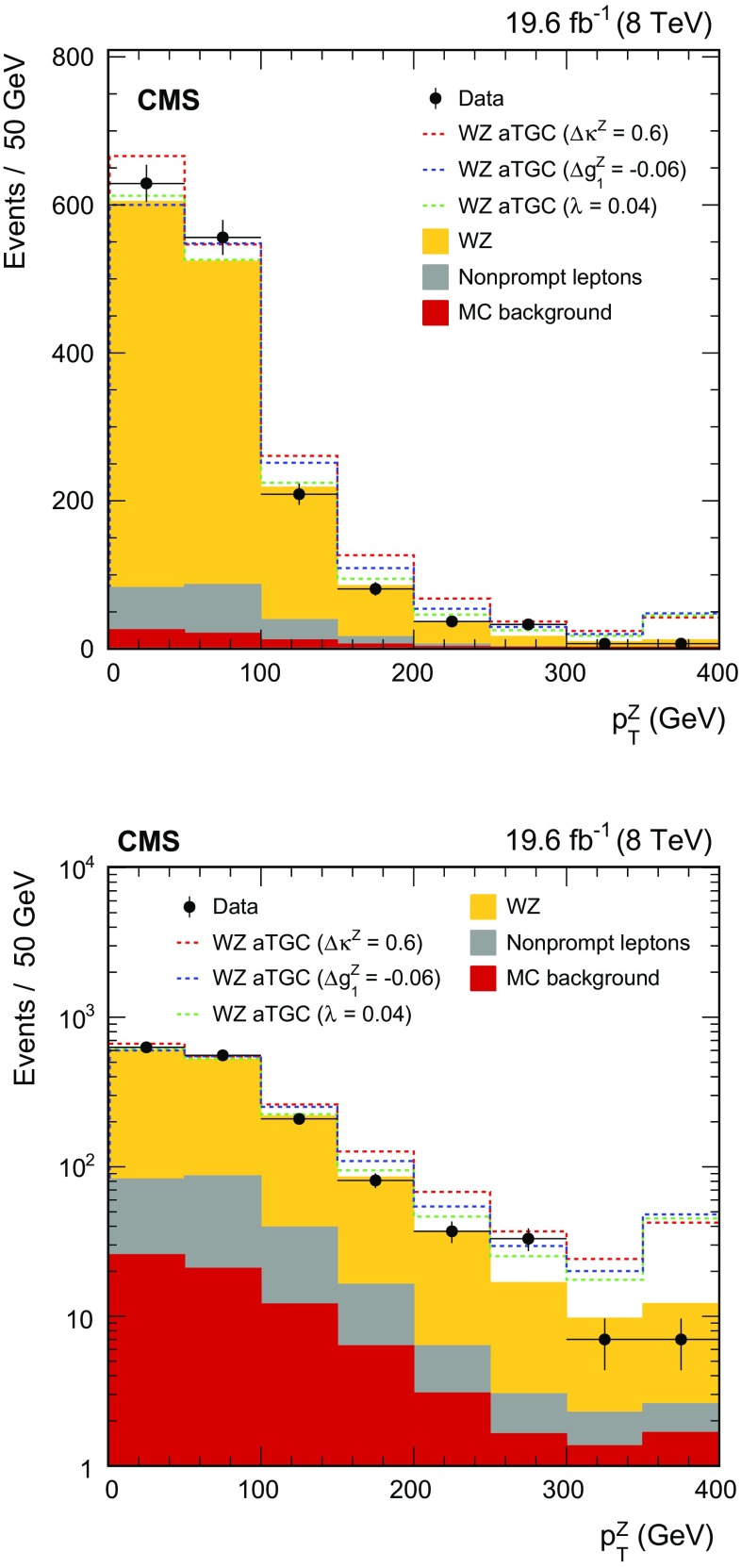
Transverse momentum distribution of the Z boson candidates, in linear scale (*top*) and log scale (*bottom*) for all channels combined. The SM WZ contribution (*light orange*) is normalized to the predicted cross section from mcfm. *Dashed lines* correspond to aTGC expectations with different parameter values. The last bin includes the integral of the tail

### Anomalous triple gauge couplings limits

Triple gauge boson couplings are a consequence of the non-Abelian nature of the SM electroweak sector. Several extensions of the SM predict additional processes with multiple bosons in the final state so any observed deviation of diboson production cross sections from their SM predictions could be an early sign of new physics. The most general Lorentz invariant effective Lagrangian that describes WWV couplings, where $\mathrm{V} = \gamma$ or Z, has 14 independent parameters [53, 54], seven for $\mathrm{V} =\gamma$ and seven for $\mathrm{V} = \mathrm{Z}$. Assuming charge conjugation (C) and parity (P) conservation, only six independent parameters remain. The effective Lagrangian, normalized by the electroweak coupling, is given by:

4 $$\begin{aligned} \frac{\mathcal {L}_{\mathrm{TGC}}}{g_{\mathrm{W}\mathrm{W}\mathrm{V}}}= & {} ig_1^{\mathrm{V}}(W^{-}_{\mu \nu } {W}^{+\mu } {V}^\nu - {W}^{-}_\mu {V}_\nu {W}^{+\mu \nu })\nonumber \\&+ i\kappa _{\mathrm{V}} {W}^{-}_\mu {W}^{+}_\nu {V}^{\mu \nu } + \frac{i\lambda _{\mathrm{V}}}{M_{\mathrm{W}}^2}W^{-}_{\delta \mu }{W}^{+\mu }_\nu {V}^{\nu \delta }, \end{aligned}$$

where ${{W^\pm }}_{\mu \nu } = \partial _\mu {W}^\pm _\nu - \partial _\nu {W}^\pm _\mu$, ${V}_{\mu \nu } = \partial _\mu V_\nu - \partial _\nu {V}_\mu$, and couplings $g_{\mathrm{W}\mathrm{W}\gamma } = -e$ and $g_{\mathrm{W}\mathrm{W}\mathrm{Z}} = -e \cot \theta _{\mathrm{W}}$, with $\theta _{\mathrm{W}}$ being the weak mixing angle. Assuming electromagnetic gauge invariance, i.e. $g_1^\gamma = 1$, the remaining parameters that describe the WWV coupling are $g_1^{\mathrm{Z}}$, $\kappa _{\mathrm{Z}}$, $\kappa _\gamma$, $\lambda _{\mathrm{Z}}$ and $\lambda _\gamma$. In the SM $\lambda _{\mathrm{Z}} = \lambda _\gamma = 0$ and $g_1^{\mathrm{Z}} = \kappa _{\mathrm{Z}} = \kappa _\gamma = 1$. The couplings are further reduced to three independent parameters if one requires the Lagrangian to be ${\mathcal {SU}}(2)_L\times {\mathcal {U}}(1)_Y$ invariant (“LEP parameterization”) [55–57]:

5 $$\begin{aligned} \Delta \kappa _{\mathrm{Z}} = \Delta g_1^{\mathrm{Z}} - \Delta \kappa _\gamma \, \tan ^2\theta _{\mathrm{W}},\quad \lambda = \lambda _\gamma = \lambda _{\mathrm{Z}}, \end{aligned}$$

where $$\Delta \kappa _{\mathrm{Z}}=\kappa _{\mathrm{Z}}-1$$, $$\Delta g_1^{\mathrm{Z}}= g_1^{\mathrm{Z}}-1$$ and $$\Delta \kappa _\gamma =\kappa _\gamma -1$$.

In this analysis we measure $\Delta \kappa _{\mathrm{Z}}$, $\lambda$, and $\Delta g_1^{\mathrm{Z}}$ from WZ production at 8$\,\text{TeV}$. No form factor scaling is used for aTGCs, as this allows us to provide results without the bias that can be caused by the choice of the form factor energy dependence.

Another approach to the parametrization of anomalous couplings is through effective field theory (EFT), with the higher-order operators added to the SM Lagrangian as follows:

6 $$\begin{aligned} \mathcal {L}_{\mathrm{EFT}} = \mathcal {L}_{ \mathrm{SM}}+\sum _{n=1}^{\infty }\sum _{i}\frac{c_{i}^{(n)}}{\Lambda ^{n}}O_{i}^{(n+4)}. \end{aligned}$$

Here $O_{i}$ are the higher-order operators, the coefficients $c_{i}$ are dimensionless, and $\Lambda$ is the mass scale of new physics. Operators are suppressed if the accessible energy is low compared to the mass scale. There are three CP-even operators that contribute to WWZ TGC, $O_{\mathrm{W}\mathrm{W}\mathrm{W}}$, $O_{\mathrm{W}}$, and $O_{\mathrm{B}}$. For the case of ‘LEP parametrization’ and no form factor scaling of aTGCs, the relations between parameters in the aTGCs and EFT approaches are as follows:

$$\begin{aligned} g_{1}^{\mathrm{Z}}= & {} 1+c_{\mathrm{W}}\frac{m_{\mathrm{Z}}^2}{2\Lambda ^2},\\ \kappa _{\gamma }= & {} 1+\left( c_{\mathrm{W}}+c_{\mathrm{B}}\right) \frac{m_{\mathrm{W}}^2}{2\Lambda ^2},\\ \kappa _{\mathrm{Z}}= & {} 1+\left( c_{\mathrm{W}}-c_{\mathrm{B}} \tan ^2\theta _{\mathrm{W}} \right) \frac{m_{\mathrm{W}}^2}{2\Lambda ^2},\\ \lambda _{\mathrm{Z}}= & {} \lambda _{\gamma } = c_{\mathrm{W}\mathrm{W}\mathrm{W}}\frac{3 g^2 m_{\mathrm{W}}^2}{2\Lambda ^2}. \end{aligned}$$

**Table 8:** One-dimensional limits on the aTGC parameters at a 95% CL for $\mathrm{W}\mathrm{Z} \rightarrow \ell \nu \ell '\ell '$

	Observed	Expected
$\Delta \kappa ^{\mathrm{Z}}$	[$-0.21, 0.25$]	[$-0.29, 0.30$]
$\Delta g_{1}^{\mathrm{Z}}$	[$-0.018, 0.035$]	[$-0.028, 0.040$]
$\lambda ^{\mathrm{Z}}$	[$-0.018, 0.016$]	[$-0.024, 0.021$]

**Table 9:** One-dimensional limits on the EFT parameters at a 95% CL for ${\mathrm{W}\mathrm{Z}}\rightarrow \ell \nu \ell '\ell '$

	Observed [$\text{TeV} ^{-2}$]	Expected [$\text{TeV} ^{-2}$]
$c_{\mathrm{B}}/\Lambda ^{2}$	[$-260, 210$]	[$-310, 300$]
$c_{\mathrm{W}}/\Lambda ^{2}$	[$-4.2, 8.0$]	[$-6.8, 9.2$]
$c_{\mathrm{W}\mathrm{W}\mathrm{W}}/\Lambda ^{2}$	[$-4.6, 4.2$]	[$-6.1, 5.6$]

**Fig. 8:**
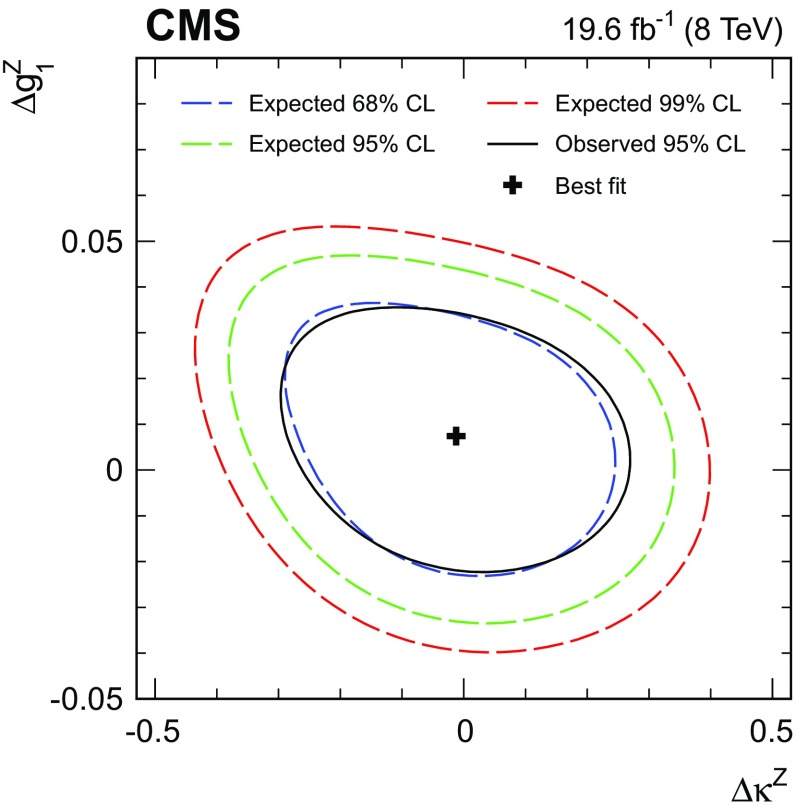
Two-dimensional observed 95% CL limits and expected 68, 95 and 99% CL limits on anomalous coupling parameters $\Delta \kappa ^{\mathrm{Z}}$ and $\Delta g_{1}^{\mathrm{Z}}$

**Fig. 9:**
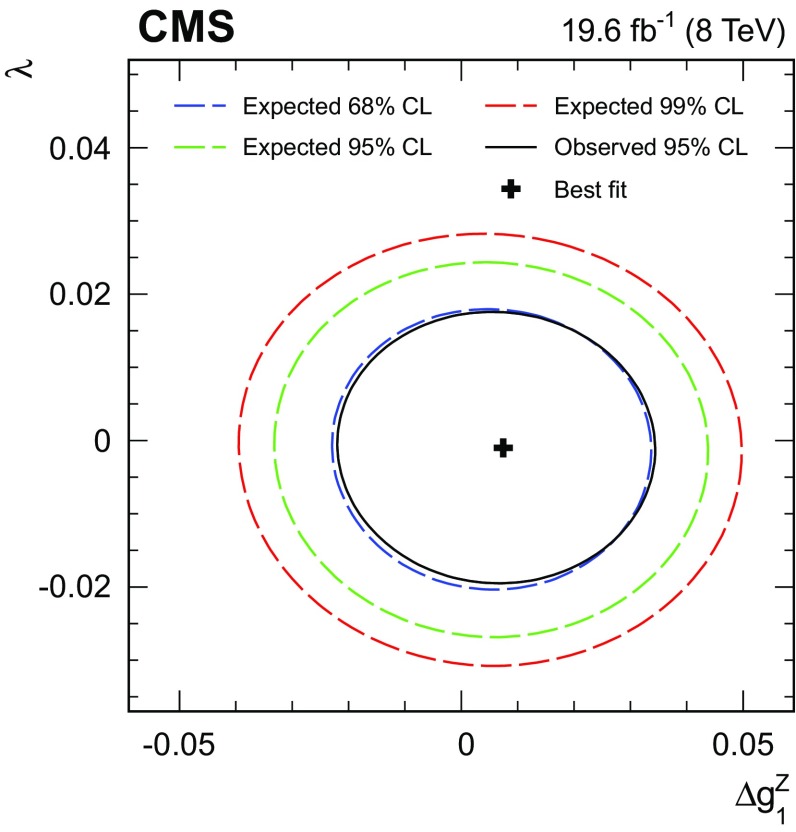
Two-dimensional observed 95% CL limits and expected 68, 95 and 99% CL limits on anomalous coupling parameters $\Delta g_{1}^{\mathrm{Z}}$ and $\lambda ^{\mathrm{Z}}$

**Fig. 10:**
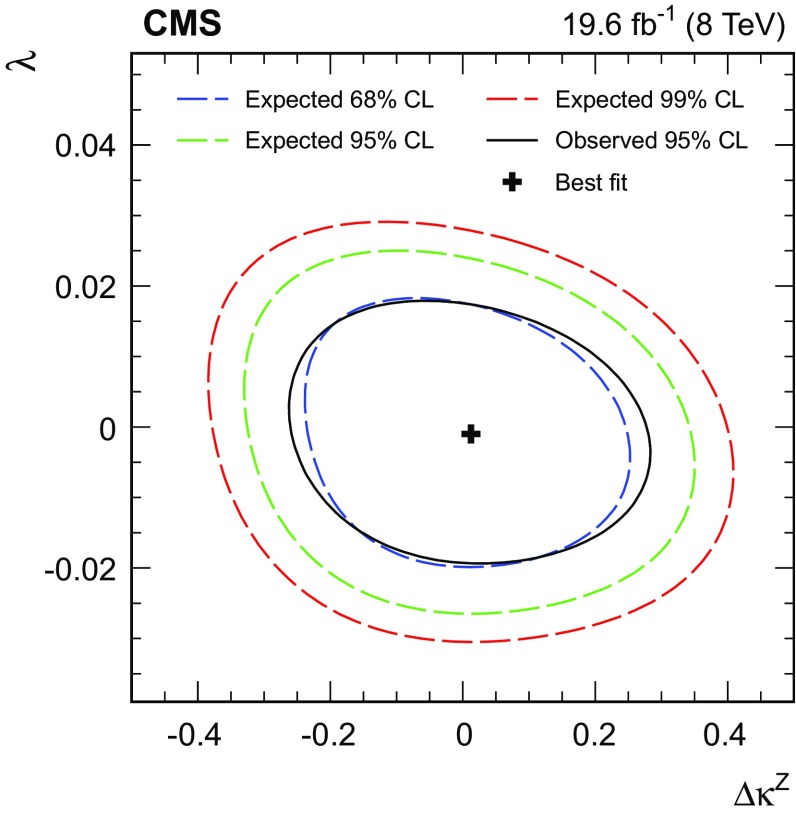
Two-dimensional observed 95% CL limits and expected 68, 95 and 99% CL limits on anomalous coupling parameters $\Delta \kappa ^{\mathrm{Z}}$ and $\lambda ^{\mathrm{Z}}$

The presence of anomalous triple gauge couplings would be manifested as an increased yield of events, with the largest increase at high Z boson transverse momentum ($p_{\mathrm{T}} ^{\mathrm{Z}}$). The expected $p_{\mathrm{T}} ^{\mathrm{Z}}$ spectrum for some aTGC values is obtained by normalizing the MadGraph events to the expected NLO SM cross section from mcfm, and then reweighting them to the expected cross section for that particular aTGC scenario, as obtained with MCFM, based on the generated value of $p_{\mathrm{T}} ^{\mathrm{Z}}$. Samples for three 2D anomalous parameter grids are generated, $\lambda$ versus $\Delta \kappa ^{\mathrm{Z}}$, $\lambda$ versus $\Delta g_{1}^{\mathrm{Z}}$, and $\Delta \kappa ^{\mathrm{Z}}$ versus $\Delta g_{1}^{\mathrm{Z}}$, where the third parameter is set to its SM value. The expected yield of the anomalous coupling signal in every $p_{\mathrm{T}} ^{\mathrm{Z}}$ bin is parametrized by a second-order polynomial as a function of two aTGC parameters for every channel. The observed $p_{\mathrm{T}} ^{\mathrm{Z}}$ spectrum is shown in Fig. 7 together with the expected spectra for a few different aTGC scenarios. A simultaneous fit to the values of aTGCs is performed [58] in all four lepton channels. A profile likelihood method, Wald gaussian approximation, and Wilks’ theorem  [59] are used to derive 1D and 2D limits at a 95% confidence level (CL) on each of the three aTGC parameters and every combination of two aTGC parameters, respectively, while all other parameters are set to their SM values. No significant deviation from the SM expectation is observed. Results can be found in Tables 8 and 9, and in Figs. 8, 9, and 10.

**Table 10:** Lowest incoming partons energy for which observed limits on the coefficients would lead to unitarity violation

	$\sqrt{s}$ [$\text{TeV}$ ]
From observed limit on $c_{\mathrm{B}}/\Lambda ^{2}$ parameter	1.6
From observed limit on $c_{\mathrm{W}}/\Lambda ^{2}$ parameter	5.1
From observed limit on $c_{\mathrm{W}\mathrm{W}\mathrm{W}}/\Lambda ^{2}$ parameter	4.3

Limits on aTGC parameters were previously set by LEP [60], ATLAS [11, 14] and CMS [15]. LHC analyses using 8$\,\text{TeV}$ data are setting most stringent limits. Results in this paper show sensitivity similar to the results given by the ATLAS Collaboration in the same channel [11].

Following the calculation in Ref. [61] we find the lowest incoming parton energy for which observed limits on the coefficients would lead to unitarity violation (Table 10). Overall, for charged aTGCs, we are in the region where unitarity is not violated.

## Summary

This paper reports measurements of the WZ inclusive cross section in proton–proton collisions at $\sqrt{s} = 7$ and 8$\,\text{TeV}$ in the fully-leptonic WZ decay modes with electrons and muons in the final state. The data samples correspond to integrated luminosities of 4.9$\,\text{fb}^{-1}$ for the 7$\,\text{TeV}$ measurement and 19.6$\,\text{fb}^{-1}$ for the 8$\,\text{TeV}$ measurement. The measured production cross sections for $71< m_{\mathrm{Z}} < 111\,\text{GeV}$ are $\sigma ({\mathrm{p}\mathrm{p}}\rightarrow {\mathrm{W}\mathrm{Z}};~\sqrt{s} = 7\,\text{TeV}) = 20.14 \pm 1.32\,\text{(stat)} \pm 0.38\,\text{(theo)} \pm 1.06\,\text{(exp)} \pm 0.44\,\text{(lumi)}$ $\,\text{pb}$ and $\sigma ({\mathrm{p}\mathrm{p}}\rightarrow {\mathrm{W}\mathrm{Z}};~\sqrt{s} = 8\,\text{TeV}) = 24.09 \pm 0.87\,\text{(stat)} \pm 0.80\,\text{(theo)} \pm 1.40\,\text{(exp)} \pm 0.63\,\text{(lumi)}$ $\,\text{pb}$. These results are consistent with standard model predictions.

Using the data collected at $\sqrt{s} = 8\,\text{TeV}$, results on differential cross sections are also presented, and a search for anomalous WWZ couplings has been performed. The following one-dimensional limits at 95% CL are obtained: $-0.21< \Delta \kappa ^{\mathrm{Z}} < 0.25$, $-0.018< \Delta g_{1}^{\mathrm{Z}} < 0.035$, and $-0.018< \lambda ^{\mathrm{Z}} < 0.016$.
